# Label-free single-cell live imaging reveals fast metabolic switch in T lymphocytes

**DOI:** 10.1091/mbc.E23-01-0009

**Published:** 2023-12-14

**Authors:** Noémie Paillon, Thi Phuong Lien Ung, Stéphanie Dogniaux, Chiara Stringari, Claire Hivroz

**Affiliations:** a Institut Curie, PSL Research University, INSERM, U932 “Integrative analysis of T cell activation” team, 75005 Paris, France; b Laboratory for Optics and Biosciences, École Polytechnique, CNRS, Inserm, Institut Polytechnique de Paris, 91128 Palaiseau, France; Cornell University

## Abstract

T-cell activation induces a metabolic switch generating energy for proliferation, survival, and functions. We used noninvasive label-free two-photon fluorescence lifetime microscopy (2P-FLIM) to map the spatial and temporal dynamics of the metabolic NAD(P)H co-enzyme during T lymphocyte activation. This provides a readout of the OXPHOS and glycolysis rates at a single-cell level. Analyzes were performed in the CD4+ leukemic T cell line Jurkat, and in human CD4+ primary T cells. Cells were activated on glass surfaces coated with activating antibodies mimicking immune synapse formation. Comparing the fraction of bound NAD(P)H between resting and activated T cells, we show that T-cell activation induces a rapid switch toward glycolysis. This occurs after 10 min and remains stable for one hour. Three-dimensional analyzes revealed that the intracellular distribution of fraction of bound NAD(P)H increases at the immune synapse in activated cells. Finally, we show that fraction of bound NAD(P)H tends to negatively correlate with spreading of activated T cells, suggesting a link between actin remodeling and metabolic changes. This study highlights that 2P-FLIM measurement of fraction of bound NAD(P)H is well suited to follow a fast metabolic switch in three dimensions, in single T lymphocytes with subcellular resolution.

## Abstract (executive summary)

Metabolic switch controls T cell differentiation and activation. Yet, the early events induced by T cell receptor (TCR) activation leading to this switch are incompletely characterized.

Using label-free two-photon fluorescence lifetime microscopy (2P-FLIM) to map the dynamics of the NAD(P)H co-enzyme in single cells and in three dimensions, we show that TCR triggering induces a rapid switch toward glycolysis concomitant with actin remodeling with a specific spatial distribution.

Our results show that this method can be used to follow spatially, in single T cells, rapid changes in the metabolism. It should provide valuable information on T cell metabolism in normal and pathological conditions.

## INTRODUCTION

T lymphocyte activation through the T-cell receptor (TCR) and coreceptors such as CD28 induces a rapid transcription of numerous new messenger RNA (mRNA) transcripts and proteins. Activated T lymphocytes also undergo massive growth, doubling to quadrupling their size in 1 to 2 days as well as several cycles of divisions (Marchingo and Cantrell, 2022). These different steps are demanding in energy and thus require a rewiring of T lymphocyte metabolism (Geltink *et al.*, 2018). Indeed, T lymphocytes change their metabolism during immune response. Briefly, naïve T cells that are metabolically quiescent mainly rely on oxidative phosphorylation (OXPHOS) for their energetic needs. Effector T cells, which are very metabolically active have higher rates of both glycolysis and OXPHOS, whereas memory T cells, which are quiescent but metabolically primed rely on fatty acid oxidation and OXPHOS (Geltink *et al.*, 2018). The field of immunometabolism has gained importance by showing that different T lymphocyte populations have different metabolic signatures, but also by unveiling that the metabolism does not only energetically supports cellular functions but also shapes them (Buck *et al.*, 2017). This better understanding of T-cell immunometabolism paved the way to harnessing metabolism for therapeutic interventions. As an example, finding the right conditions to generate CAR T cells that are “metabolically fit” (Irving *et al.*, 2017) or overcoming metabolic competition in the tumor’s microenvironment to improve antitumoral T-cell responses (Corrado and Pearce, 2022). Thus, there is an increasing need to better understand immunometabolism in T lymphocytes.

Many tools have been developed to measure cell metabolism and have been used in T lymphocytes. Among them, 13 C-based stable isotope labeling (SIL) techniques facilitate tracing the metabolic fate of nutrients in cells (Jang *et al.*, 2018), the Seahorse Extracellular Flux Analyzer simultaneously measures, in real-time, the extracellular acidification rate (ECAR; an indicator of glycolysis) and the oxygen consumption rate (OCR; an indicator of OXPHOS from relatively low numbers of cells. Both methods allow the metabolic analysis of cells in bulk. Two other recent elegant assays were developed recently: SCENITH and SPICE-Met respectively rely on characterizing the energetic metabolism profile of cells by monitoring changes in protein synthesis levels in response to metabolic inhibitors (Argüello *et al.*, 2020), and on sensing the ATP:ADP ratio in cells expressing the genetically encoded fluorescent biosensor (Tantama *et al.*, 2013). The SCENITH assay, based on flow cytometry, monitors the metabolic profile at a single cell resolution but not in real time and has been used successfully in T cells (Lopes *et al.*, 2021). The SPICE-Met assay is based on one or two-photon microscopy, allows to follow single cells with real-time imaging, has been used successfully in T cells (Russo *et al.*, 2022) but requires the expression of a transgene.

Most of the studies aiming at analyzing metabolism in T lymphocytes have been performed in cell populations at steady state or several hours after exposure to activation signals. Rapid changes (in the order of 30 to 60 min) in OXPHOS and aerobic glycolysis have yet been reported upon activation of T lymphocytes (Menk *et al.*, 2018; Walsh *et al.*, 2020).

To follow the metabolic profiles overtime in activated T cells, we used two-photon fluorescence lifetime microscopy (2P-FLIM) of the coenzyme NAD(P)H, as it reports metabolic processes in a label-free and noninvasive manner (Skala *et al.*, 2007; Stringari *et al.*, 2011; Datta *et al.*, 2020). NAD(P)H fluorescence lifetime is exquisitely sensitive to enzyme binding during the cycling of the electron transport chain, the protein-bound NAD(P)H lifetime being significantly longer than the free NAD(P)H lifetime. Both this particularity and its ubiquity render NAD(P)H one of the most useful and informative intrinsic biomarkers for metabolism in live cells and tissues (Zipfel *et al.*, 2003; Heikal, 2010). Through the readout of protein-bound and free NAD(P)H, 2P-FLIM provides sensitive measurements of the redox states (NAD^+^/ NAD(P)H) of cells as well as OXPHOS and glycolysis rates (Lakowicz *et al.*, 1992; Bird *et al.*, 2005; Vishwasrao *et al.*, 2005; Skala *et al.*, 2007; Stringari *et al.*, 2011, 2012; Ung *et al.*, 2021). 2P-FLIM has the spatiotemporal resolution required to characterize NAD(P)H subcellular compartments (Sánchez-Ramírez *et al.*, 2022) and to measure metabolic shifts induced upon T lymphocyte activation (Walsh *et al.*, 2020) as well as metabolic polarization and heterogeneity in macrophages and microglia in vitro (Alfonso-García *et al.*, 2016; Abdul *et al.*, 2020; Smokelin *et al.*, 2020) and in vivo (Bernier *et al.*, 2020; Heaster *et al.*, 2020, 2021; Miskolci *et al.*, 2022). In the present study we used 2P-FLIM of NAD(P)H to follow the dynamic of NAD^+^/NAD(P)H redox ratio (through the fraction of bound NAD(P)H) in Jurkat T cells (a leukemic T-cell line) and primary human CD4^+^ T lymphocytes, during activation. We activated the cells on glass slides coated with anti-CD3 + anti-CD28 antibodies, which induce polarization and spreading of T cells and mimic the immune synapse formation (Bunnell *et al.*, 2001). Our results show that T-cell activation induces a rapid decrease of fraction of bound NAD(P)H, reflecting a decrease in OXPHOS/glycolysis ratio in activated T lymphocytes. This can be followed, upon time, at a single-cell level and in 3D. The three-dimensional metabolic maps suggest that the distribution of fraction of bound NAD(P)H in the cell is changing in different cellular compartments upon immune synapse formation. Moreover, because remodeling of the actin cytoskeleton is one of the early hallmarks of T-cell activation, we expressed LifeAct-mCherry, a 17-amino-acid peptide, which stains filamentous actin (F-actin) structures, in Jurkat T cells and followed the bound/free NAD(P)H ratio simultaneously with the spreading area. This analysis shows that the fraction of bound NAD(P)H diminished in spreading cells suggesting that there might be a link between actin remodeling in T cells and metabolic changes. Together, our data show that upon activation mimicking immune synapse formation, T lymphocytes dynamically modify their metabolism in time and space.

## RESULTS

### Two-photon FLIM of the metabolic coenzyme NAD(P)H in T lymphocytes

In this study, we implemented longitudinal label-free 2P-FLIM (Skala *et al.*, 2007; Stringari *et al.*, 2011) in live T lymphocytes to create noninvasive metabolic maps of the metabolic coenzyme NAD(P)H at a submicron resolution and measured the spatial and temporal dynamics of redox metabolism during T lymphocyte activation.

**FIGURE 1:**
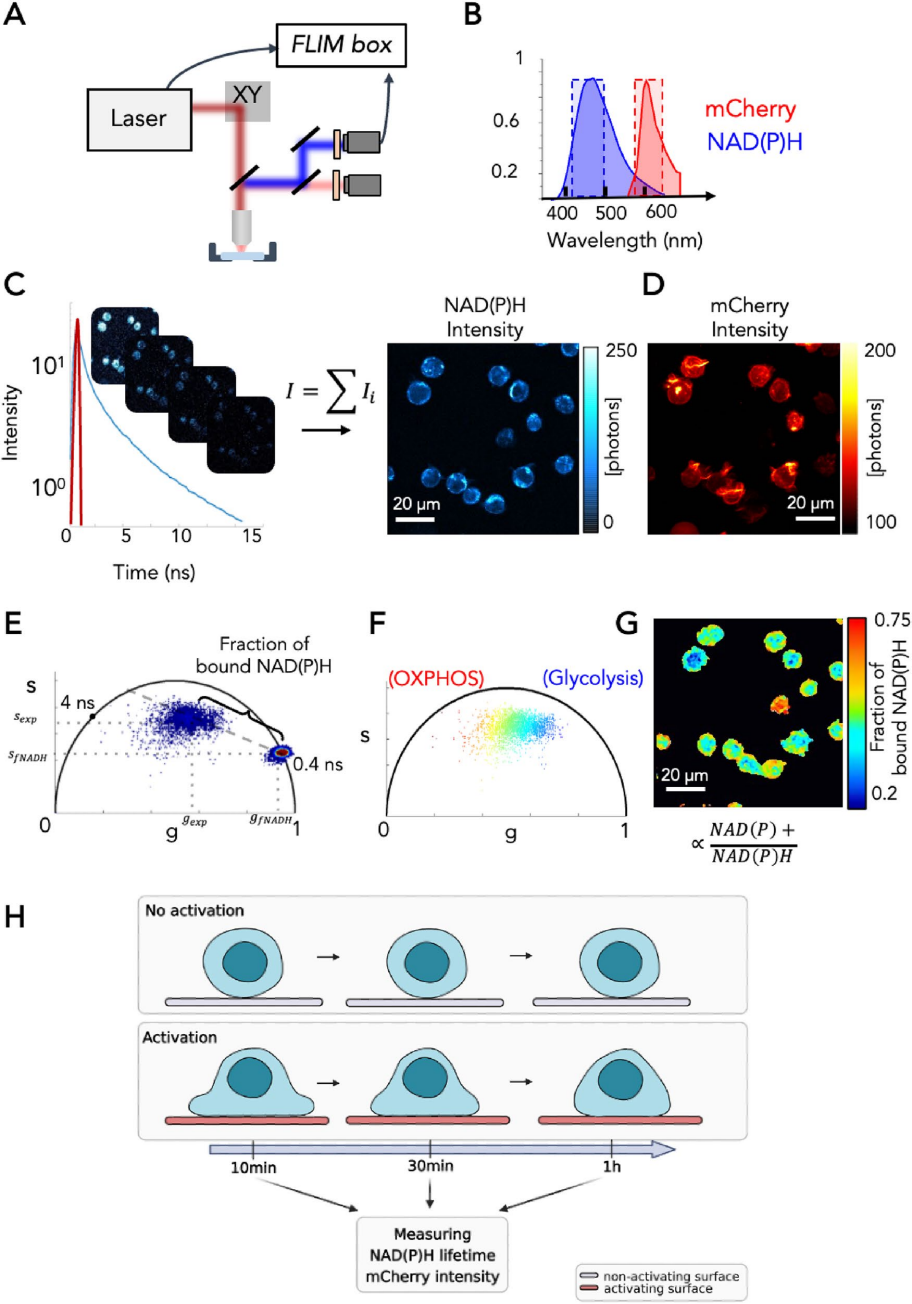
Experimental setup of two-photon excitation FLIM*.* (A) Scheme of the experimental setup used for this work. (B) Emission fluorescence of NAD(P)H and LifeAct-mCherry were collected with band-pass filters 460/80 nm and 607/70 nm, respectively. (C) Each fluorescence lifetime image contains a fluorescence lifetime decay in every pixel. The NAD(P)H intensity image is determined by the sum of the FLIM stack. (D) LifeAct-mCherry intensity image simultaneously acquired in the red channel. (E) The multiexponential fluorescence intensity decay in every pixel of the image is transformed in the phasor plot by fast Fourier transform. Phasor plot is color mapped as a histogram showing the density of points in phasor location. The estimation of the fraction of bound NAD(P)H is performed by measuring the distance of every experimental pixel from the location of free NAD(P)H. (F) Phasor plot color mapped with the fraction of bound NAD(P)H contrast. Blue pixels in the right part of the phasor plot have low fraction of bound NAD(P)H values with a more glycolytic phenotype, while red pixels within the left part of the phasor plot have high fraction of bound NAD(P)H values and a more OXPHOS phenotype. (G) FLIM image color mapped with fraction of bound NAD(P)H contrast (proportional to the redox ratio NAD(P)+/NAD(P)H ratio). (H) Schematic representation of the experimental setup of T-cell activation.

The principle of 2P-FLIM NAD(P)H is described in Figure 1. To obtain excitation of NAD(P)H and LifeAct-mCherry simultaneously, we excited the T lymphocytes in culture with an excitation wavelength of 740nm, while the emitted fluorescence was collected in the blue and red spectral ranges (Figure 1, A and B; *Material and Methods*). We determined the minimal illumination conditions permitting longitudinal 2P-FLIM of NAD(P)H with a good signal to noise ratio and with negligible photoperturbation (*Material and Methods*; Supplemental Figure S1). Representative NAD(P)H two-photon (2P) intensity images of live Jurkat T lymphocytes and simultaneously acquired 2P LifeAct-mCherry intensity images are shown in Figure 1, C and D. We performed FLIM of NAD(P)H in T lymphocytes using a commercial time-correlated single photon counting (TCSPC) system and we established experimental conditions that allow noninvasive longitudinal metabolic imaging of the same cells without any photodamage (Figure 1C; *Materials and Methods*). The fluorescence lifetime of NAD(P)H changes upon binding to enzymes within the electron transport chain. Indeed, the protein-bound NAD(P)H lifetime is significantly longer than the free NAD(P)H lifetime, due to self-quenching in the free state (Datta *et al.*, 2020). Thus, FLIM is used to provide sensitive measurements of the free and protein-bound NAD(P)H ratio to estimate the contribution of glycolysis versus OXPHOS in ATP production (Zipfel *et al.*, 2003; Skala *et al.*, 2007; Heikal, 2010). The NAD(P)H FLIM images were analyzed through phasor analysis (Stringari *et al.*, 2011; Becker, 2012; Figure 1E). To quantify the subcellular metabolism, we estimated the fraction of bound NAD(P)H in every pixel of the image by calculating the distance between the experimental point in the phasor plot and the location of free NAD(P)H (Figure 1E; *Material and Methods*). The phasor plot (Figure 1F) and the FLIM image (Figure 1G) are color mapped with the fraction of bound NAD(P)H contrast which is proportional to the redox ratio NAD(P)+/ NAD(P)H and the OXPHOS/glycolysis ratio (Bird *et al.*, 2005; see *Material and Methods*). The fraction of bound NAD(P)H map (Figure 1G) highlights the metabolic heterogeneity among cells and the subcellular NAD(P)H compartmentalization. This experimental setup was applied to establish the cellular metabolic trajectory of human T lymphocytes in resting and activating conditions.

### Longitudinal FLIM imaging of the metabolic coenzymes NAD(P)H in resting and activated Jurkat T cells overtime

**FIGURE 2:**
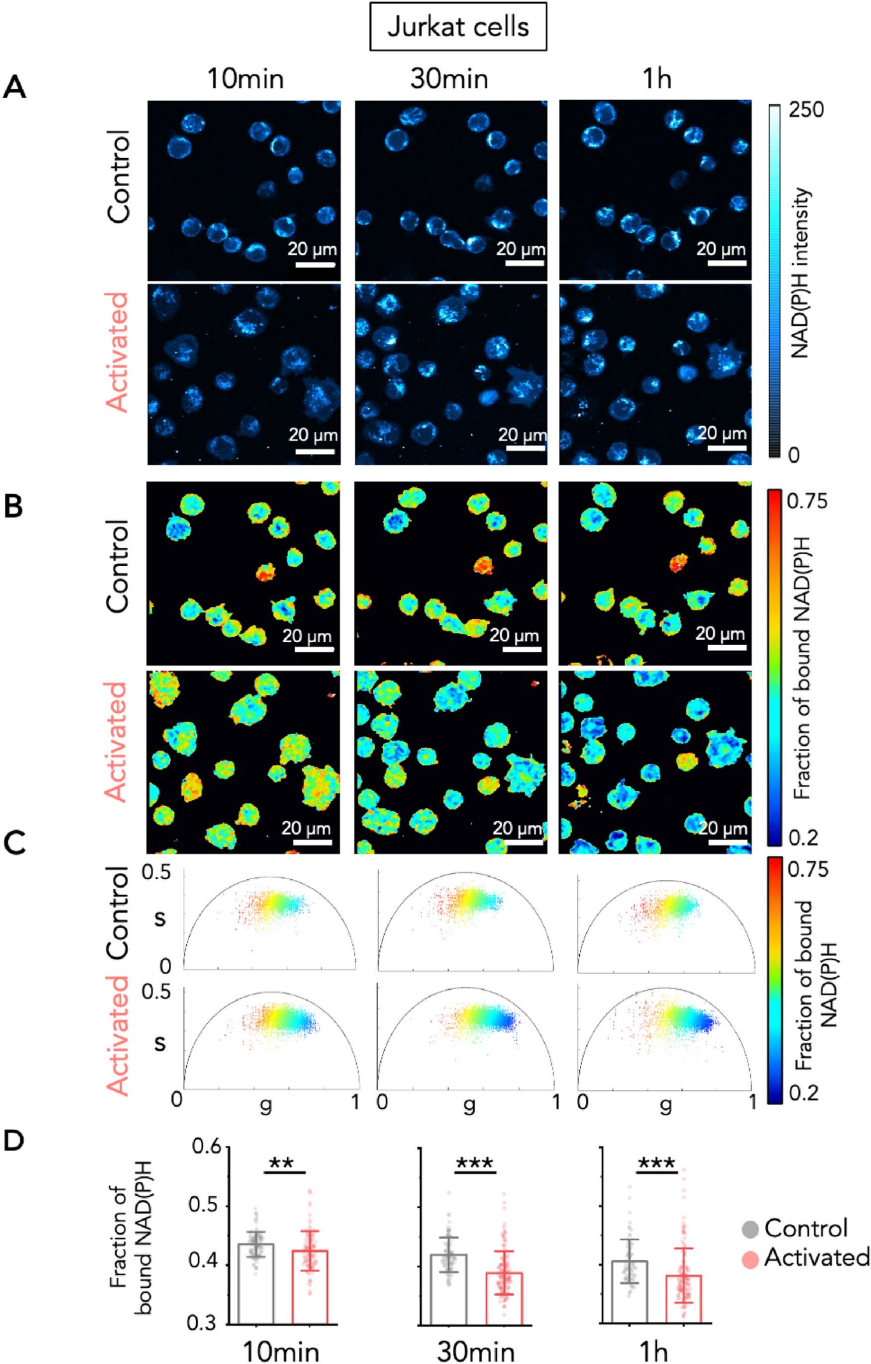
Metabolic shift during Jurkat T-cell activation revealed by NAD(P)H FLIM. (A–D) Jurkat T cells were incubated on glass surfaces coated with poly-L-lysine alone (control), or poly-L-lysine with anti-CD3/CD28 antibodies (activated). For each condition, mages of three different regions of interest were acquired at three time points (10 min, 30 min, and 1 h). (A) Representative images of NAD(P)H intensity, (B) maps of fraction of bound NAD(P)H and (C) phasor plots of Jurkat T cells. Each point in the phasor plot is color coded following the fraction of bound NAD(P)H. (D) Fraction of bound NAD(P)H measured in Jurkat T cells in control or activated condition at the different timepoints. *T* test, **P ≤ 0.01, ***P < 0.001. Data are presented as median with interquartile range.

**FIGURE 3:**
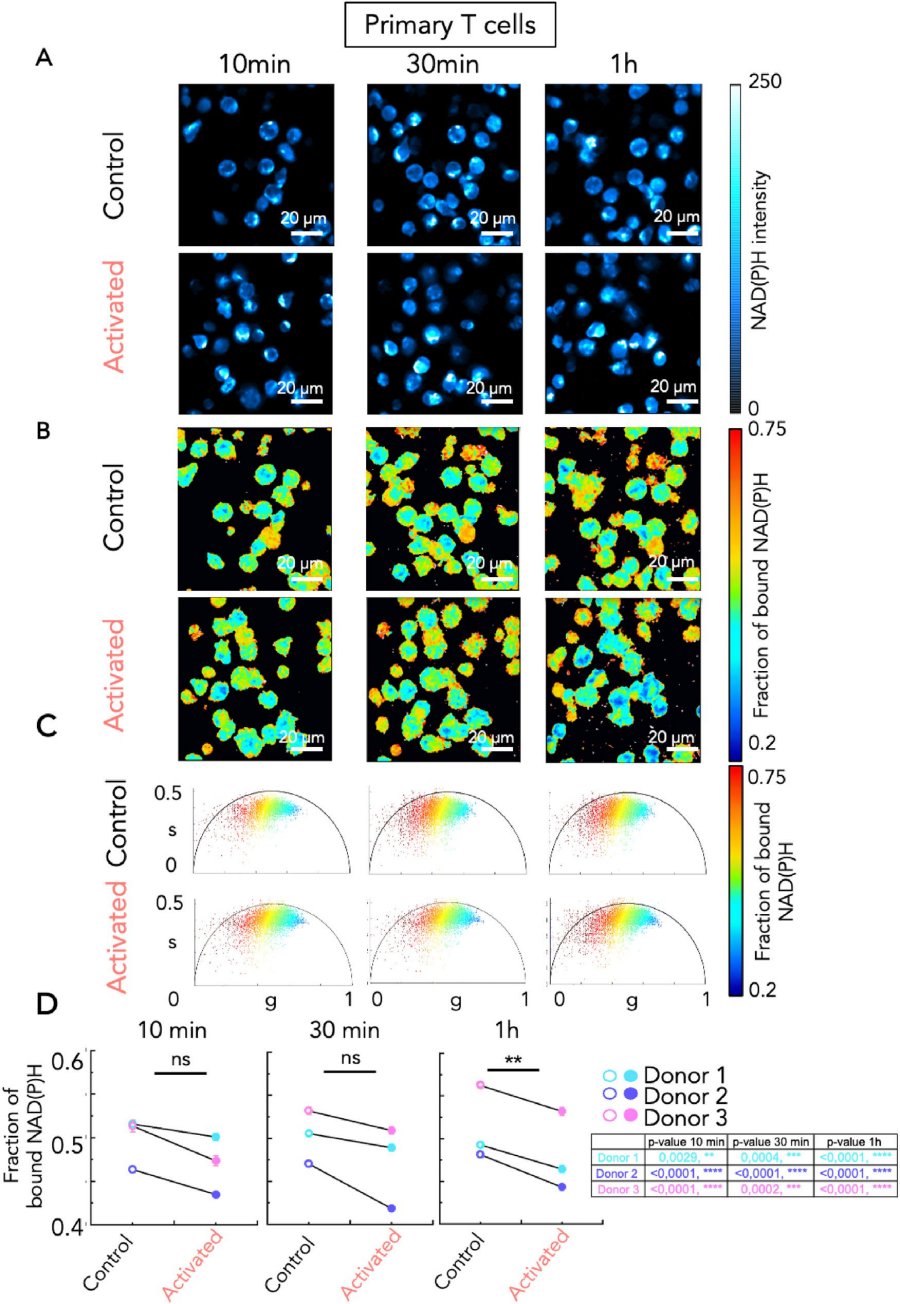
Metabolic shift during primary T-cell activation revealed by NAD(P)H FLIM. A-D. Human primary CD4+ T cells were incubated on glass surfaces coated with poly-L-lysine alone (control), or poly-L-lysine with anti-CD3/CD28 antibodies (activated). For each condition, three different regions of interest were acquired at three time points (10 min, 30 min, and 1 h). (A) Representative images of NAD(P)H intensity, (B) maps of fraction of bound NAD(P)H and (C) phasor plots of primary T cells. Each point in the phasor plot is color coded following the fraction of bound NAD(P)H. (D) Fraction of bound NAD(P)H measured in primary T cells in control or activated condition at the different time points. N = 3 independent experiments with three different healthy donors. Data presented on the left panel are means for each donor, data presented in the table are the *p* values from the paired *T* test done on each donor between control and activated condition. *T* test, **P ≤ 0.01.

The aim of the study was to measure the temporal dynamics of fluorescence lifetime of NAD(P)H in T cells after activation, to determine the glycolytic capacity of T cells in real time. To do so, we used a model that has been developed to mimic activation of T cells at the immune synapse. Cells were dropped on glass surfaces coated with either poly-L-Lysine (PLL) alone, or PLL and anti-CD3 + anti-CD28 activating antibodies (Figure 1H). This activation is known to induce early signaling and is accompanied by spreading of T cells on the surface. Measurements were first performed on Jurkat cells in 3D at 10, 30, and 60 min after plating the cells on PLL or activating antibodies (Figure 1H). We first used Jurkat T cells transduced with a lentiviral vector encoding LifeAct-mCherry. To obtain a precise analysis of the intracellular metabolic state, we performed analyzes both at the pixel level and at the single-cell level by considering the metabolic signature of the entire cell in 3D and averaging the fraction of bound NAD(P)H inside the mask defined by the LifeAct-mCherry fluorescence (Supplemental Figure S2B). The mean value of the fraction of bound NAD(P)H of single cells in each Z plane was determined (Supplemental Figure S2C) by averaging the fraction of bound NAD(P)H over the segmented region of interest of the single cells in each Z plane. We could thus follow in time the NAD(P)H intensity and fraction of bound NAD(P)H in each single cell longitudinally. Examples of three Jurkat T cells in the same field of view in resting (1 to 3) and activating (4 to 6) conditions are represented (Supplemental Figure S3). In the control, nonactivated Jurkat cells plated on PLL, the distribution of the NAD(P)H two-photon fluorescence intensity presented a dim central area, corresponding to nuclei and a brighter cortical area reflecting the presence of mitochondria in the cytosol (Figure 2A, upper raw). In fact, mitochondria have significantly higher NAD(P)H/NAD+ ratio (1:5 to 1:10) with respect to the nucleus and cytoplasm (1:400 to 1:700) (Lakowicz *et al.*, 1992; Bird *et al.*, 2005) and NAD(P)H is fluorescent while NAD^+^ is not. The fraction of bound NAD(P)H in nonactivated and in activated Jurkat cells decreased over time (Supplemental Figure S4). Yet, comparison of the fraction of bound NAD(P)H, between nonactivated and activated Jurkat cells, revealed a significant decrease of the fraction of bound NAD(P)H in activated Jurkat cells at each time point analyzed (Figure 2). We also estimated the lifetime of NAD(P)H in terms of the phase (TauP) or the modulation (TauM) as described previously (Stringari *et al.*, 2017), showing a significant NAD(P)H lifetime decrease in activated Jurkat cells (Supplemental Figure S5). We note that in this paper we chose to plot the fraction of bound NAD(P)H because it is proportional to the redox ratio NAD(P)+/NAD(P)H. We then performed the same analysis, at the single cell level, on primary human activated CD4+ helper T cells. Since these cells did not express LifeAct-mCherry, segmentation of the cells was made on the NAD(P)H intensity images. Results were comparable to what was observed in Jurkat T cells. Activation of primary T cells by CD3+CD28 activating antibodies induced a significant decrease in fraction of bound NAD(P)H at each time point and for each donor tested (Figure 3). Analysis of NAD(P)H lifetime also showed a significant decrease in activated primary T cells (Supplemental Figure S6).

Altogether these data suggest that anti-CD3+CD28 activation of Jurkat T cells and primary human CD4^+^ T cells induces a rapid shift (from 10 min) along the metabolic trajectory from OXPHOS to glycolysis. To confirm that the metabolism in the different conditions is reflected by the fraction of bound NAD(P)H in T cells, we treated the cells with rotenone, which is a drug inhibiting the complex I of the mitochondrial electron transport chain, thus blocking OXPHOS (Supplemental Figure S7). As shown in Supplemental Figure S7, rotenone treatment in resting Jurkat T cells significantly decreased the fraction of bound NAD(P)H, reflecting a shift from OXPHOS to glycolysis in the treated T cells. Conversely, treatment of activated Jurkat T cells with 2-deoxy-d-glucose (2-DG), which interferes with d-glucose metabolism, induced a strong increase in fraction of bound NAD(P)H (Supplemental Figure S7) reflecting the inhibition of glycolysis. In primary T cells, rotenone also induced a decrease in the fraction of bound NAD(P)H in nonactivated and activated T cells, whereas 2-DG induced an increase of the fraction of bound NAD(P)H in both resting and activated cells (Supplemental Figure S8). Altogether, these results suggest that T-cell activation induces a switch from OXPHOS to glycolysis, that is rapid (in the order of minutes) and remains stable for at least an hour.

### 3D analysis of the fraction of bound NAD(P)H in Jurkat T cells

**FIGURE 4:**
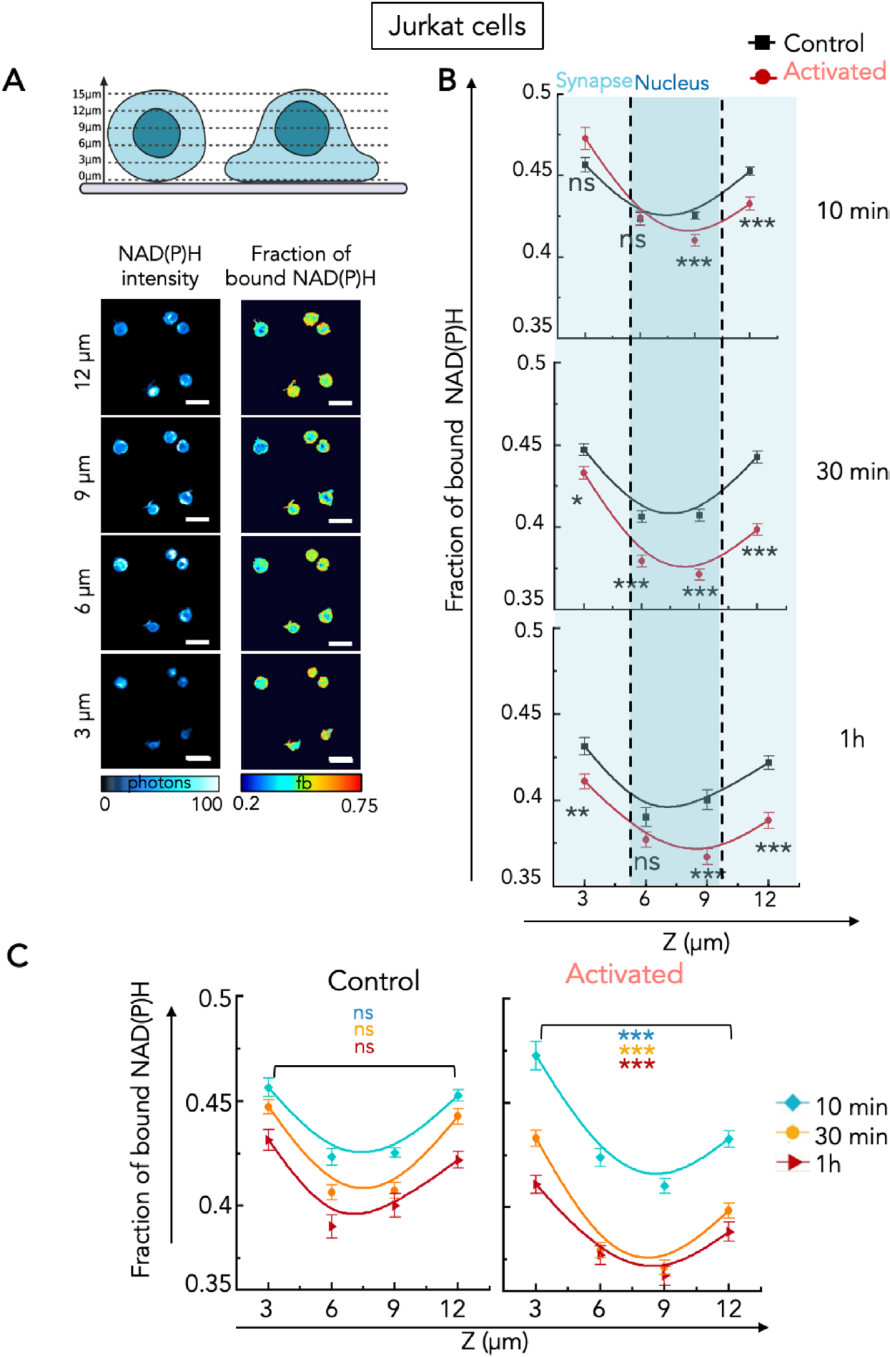
Three-dimensional analysis of the fraction of bound NAD(P)H in control and activated Jurkat cells. (A) Schematic representation of three-dimensional imaging performed in T cells and representative images of NAD(P)H intensity and fraction of bound NAD(P)H of single Jurkat T cells in the control condition at 10 min in different Z planes. (B–C) Quantification of fraction of bound NAD(P)H in control and activated conditions in every Z plane of the cells at three time points: 10 min, 30 min, and 1 h. (B) Comparison of the intracellular distribution of the fraction of bound NAD(P)H between nonactivated (control) and activated Jurkat T cells at each time point. Data are presented as mean with SD range. Statistics are indicated for each timepoint, and for the four Z planes analyzed. (C) Comparison of the fraction of bound NAD(P)H between bottom (3 µm) and top (12 µm) of the cell in nonactivated (control) and activated Jurkat T cells. Statistics between Z = 3 µm and Z = 12 µm are indicated for each time point in a different color. Data are presented as mean with SD range. Connection line is presented following the Bezier spline (B-Spline) connection in OriginPro2023 (OriginLab,Massachusetts, USA). *T* test: **P ≤ 0.01, *** *P* ≤ 0.001, ns: nonsignificant.

As described previously (Supplemental Figure S2), the analysis of the fraction of bound NAD(P)H was performed both in time and space, at different Z planes of single T cells. We studied the three-dimensional distribution of the intracellular fraction of bound NAD(P)H in resting and activated conditions at the three chosen time points. As shown in representative images (Figure 4A) and analysis of the intracellular fraction of bound NAD(P)H distribution in 700 Jurkat T cells (Figure 4B), the fraction of bound NAD(P)H distribution showed a characteristic distribution in both quiescent and activated T cells. In both cases, the two highest values of fraction of bound NAD(P)H were found at the bottom (the contact zone with the slide: 3 μm) and top of the cells (12 μm), where mitochondria are present (see representative cells in Supplemental Figure S9), while the central zone, where the nucleus is predominant (Supplemental Figure S9), presents the lowest values of fraction of bound NAD(P)H (Figure 4B). Moreover, for most Z planes, the fraction of bound NAD(P)H in activated Jurkat T cells was lower than the fraction of bound NAD(P)H in resting cells (Figure 4B), confirming the results obtained by averaging the fraction bound values of all Z planes (Figure 3) and suggesting no specific cellular compartmentalization of the activity. The difference between fraction of bound NAD(P)H in resting and activated T cells was accentuated over time (Figure 4B) in every location of the cell, showing that the shift toward a glycolytic activity, which starts a few minutes after CD3/CD28 activation, is steady. The distribution of fraction of bound NAD(P)H in the activated cells (Figure 4C, Activated) shows asymmetry between the cell side closer to the slide (the surrogate immune synapse) and its opposite, with a fraction of bound NAD(P)H that is higher at the immune synapse than in the top of the cells. This asymmetry suggests that there is relatively more OXPHOS at the synaptic side than in the rest of the cell, whereas in resting cells the distribution of fraction of bound NAD(P)H is symmetric (Figure 4C, control). This might be due to the polarized distribution of mitochondria at the immune synapse that was reported (Quintana *et al.*, 2007; Baixauli *et al.*, 2011; He *et al.*, 2019) and that we observed in our model (Supplemental Figure S9).

Altogether, these data show that 2P-FLIM of NAD(P)H in T cells can reveal temporal and spatial dynamic changes in their metabolism at a single-cell level and in subcellular compartments.

### Following T-cell spreading at the immune synapse together with the fraction of bound NAD(P)H

**FIGURE 5:**
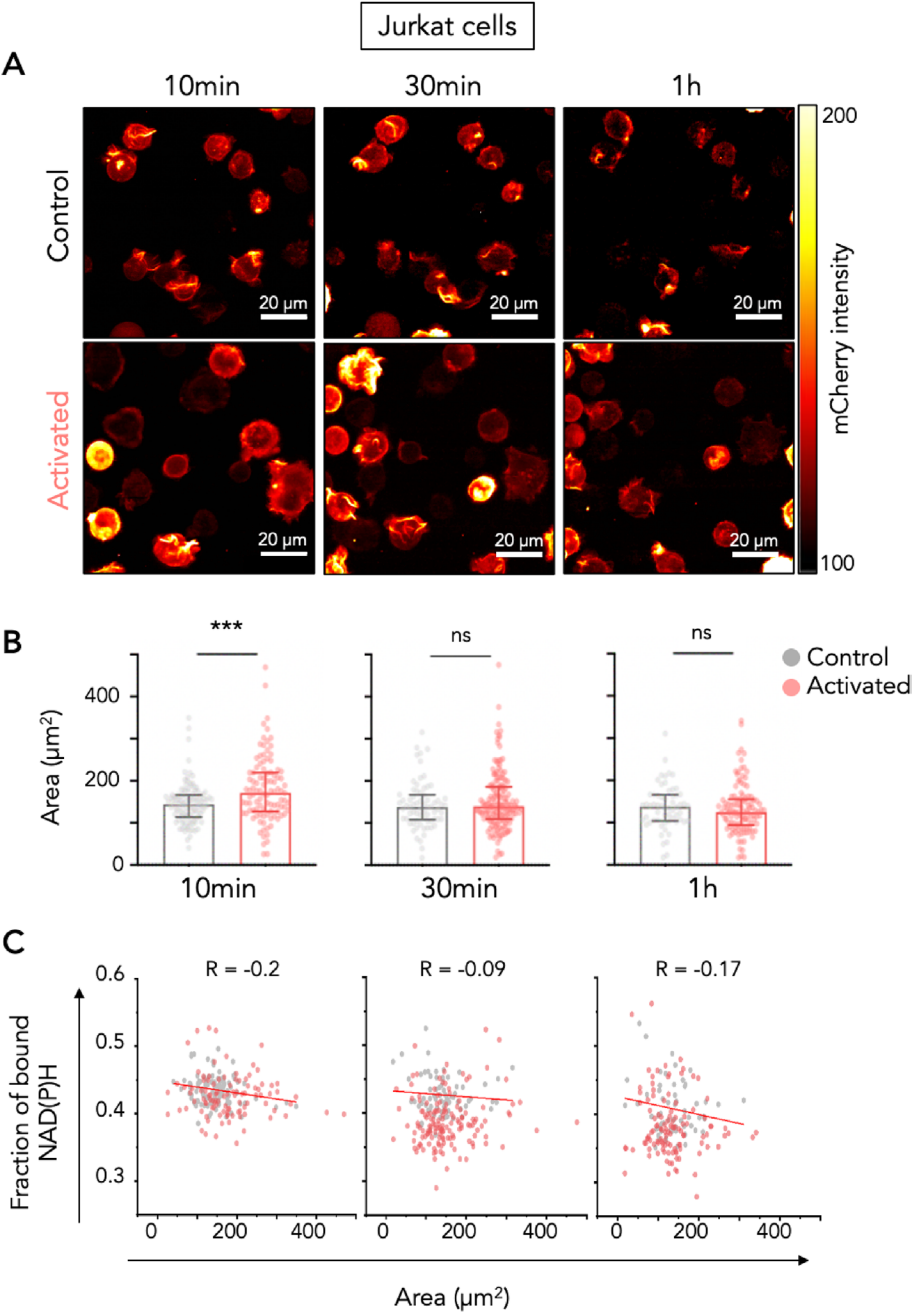
Simultaneous analysis of Jurkat T-cell spreading and metabolic shift during activation. (A–C) The cells were incubated on glass surfaces coated with poly-L-lysine alone (control), or poly-L-lysine with anti-CD3/CD28 antibodies (activated) and images of three different regions of interest were acquired at the following time points: 10 min, 30 min, and 1 h. (A) Representative images of LifeAct-mCherry intensity of Jurkat T cells in control and activated condition over time (at 10 min, 30 min, and 1 h). (B) Quantification of the cell area over time (10 min, 30 min, and 1 h) in control and activated Jurkat T cells. Area of each cell was determined on the LifeAct-mCherry channel, by making a binary mask defining each cell’s size. Data is presented by median with interquartile range, with each dot representing one measured cell. *T* test: *** *P* ≤ 0.001, ns = nonsignificant. (C) Correlation plot between the measured areas and the fraction of bound NAD(P)H of control (gray dots) and activated (red dots) T cells over time (10 min, 30 min, and 1 h).

To better follow the shape of the T cells during activation and define the binary cell mask described in Supplemental Figure S2B, we transduced Jurkat T cells with LifeAct-mCherry that labels filamentous actin (F-actin). Within minutes of contact with an activating surface, the actin networks are dynamically and drastically remodeled^31^ forming a bright F-actin ring at the cell periphery (Figure 5A).

**FIGURE 6:**
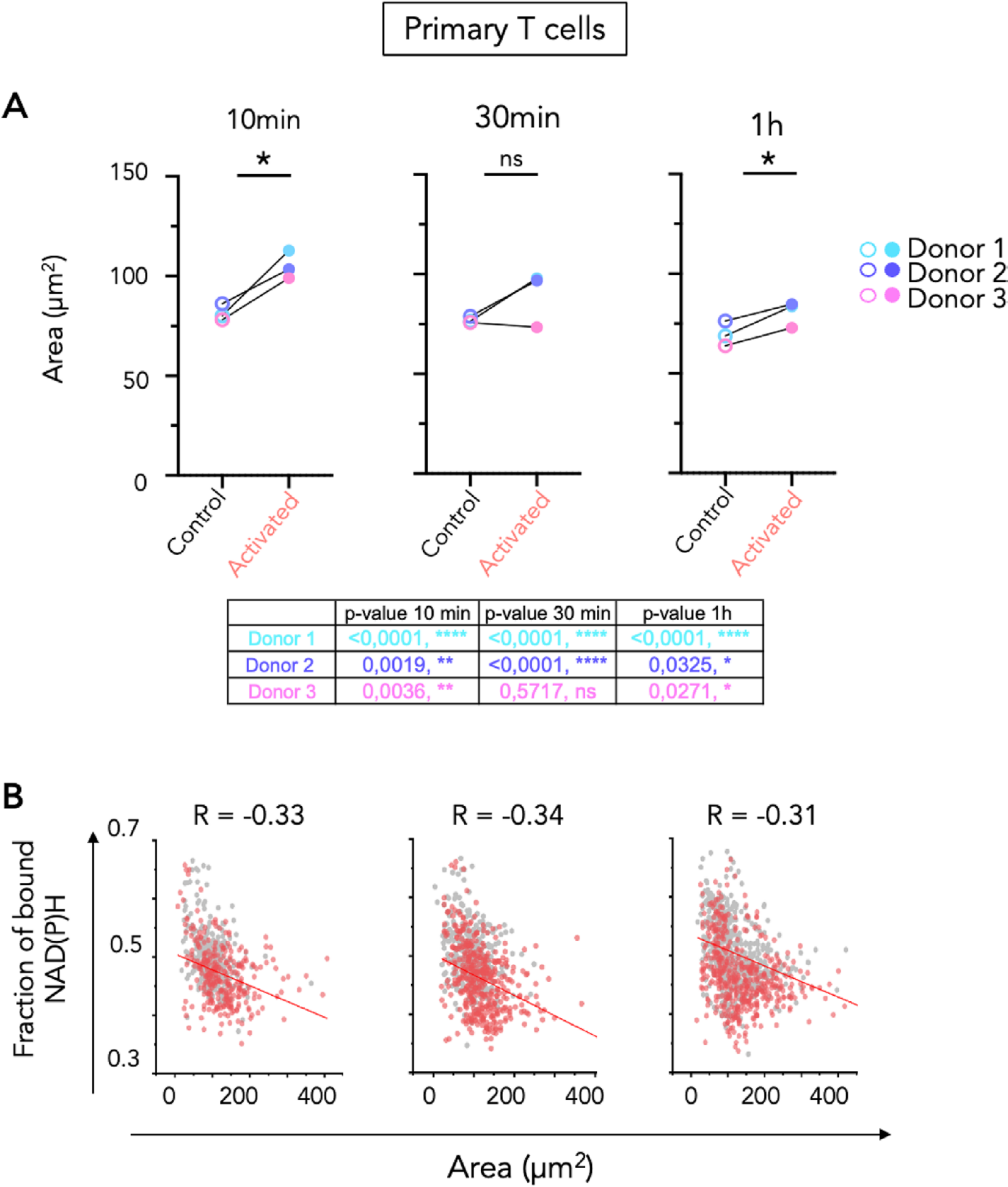
Simultaneous analysis of primary T-cell spreading and metabolic shift during activation. The primary cells from three donors were incubated on glass surfaces coated with poly-L-lysine alone (control), or poly-L-lysine with anti-CD3/CD28 antibodies (activated) and images of three different regions of interest were acquired at the following time points: 10 min, 30 min, and 1 h. N = 3 independent experiments with three different healthy donors. (A) Quantification of primary T cell area over time (10 min, 30 min, and 1 h) in control and activated T cells. The area of each cell was determined on the NAD(P)H intensity channel, by making a binary mask defining each cell’s size. Each dot represents the mean of the measured cells for each donor. Data presented in the table are the *p* values from the paired *T* test done on each donor between control and activated condition. *T* test: * *P* ≤ 0.05. (B) Correlation plot between the measured areas and fraction of bound NAD(P)H of control (grey dots) and activated (red dots) T cells over time (10 min, 30 min, and 1 h). Every point represents the mean of single cells from the three donors.

We thus simultaneously measured in 3D the spreading on the control (Poly-l-Lysine) or activating surface (anti-CD3+anti-CD28) and the fraction of bound NAD(P)H in the same cell. As shown in representative images in Figure 5A and quantification in Figure 5B, activation of Jurkat T cells induced a spreading of T cells on the activating surface at 10 min. At later time points, cells retracted as previously described (Ueda *et al.*, 2011).To investigate the relationships that may exist between actin remodeling at the immune synapse and changes in T-cell metabolism, we plotted fraction of bound NAD(P)H as a function of cell area for all the cells (control and activated conditions) at the three time points. As shown in Figure 5C, our data show that spreading Jurkat cells tend to show lower fraction of bound NAD(P)H, which suggests that cells remodeling their actin cytoskeleton the most actively are more glycolytic. This is more striking at 10 min, when cells are spreading the most. We performed the same analysis in human primary T cells from the three donors presented in Figure 3. In this case, the NAD(P)H intensity was used to draw the mask of each cell. Spreading of primary T cells was more stable overtime than spreading of Jurkat T cells, and the area of primary T cells plated on activating antibodies was always significantly bigger than the area on cells plated on Poly-L-Lysine alone (Figure 6A). Moreover, there was a more convincing negative correlation between fraction of bound NAD(P)H and area of the primary T cell at all time points tested (Figure 6B and data from individual donor in Supplemental Figure S10). Altogether, these results show that FLIM of NAD(P)H in T cells can reveal dynamic changes in their metabolism at a single-cell level and can be combined with the analysis of other parameters, such as actin remodeling, that can be followed by fluorescence imaging.

## DISCUSSION

Metabolic responses in T cells are highly dynamic and heterogeneous both temporally and spatially, which make them difficult to analyze. Therefore, quantitative, high-resolution, label-free methods to noninvasively examine metabolic processes in cells are particularly needed to better characterize and elucidate the role of different metabolic pathways in normal and pathophysiological conditions. Two-photon excited fluorescence is well appropriated for sensitive, quantitative, label-free, high-resolution assessments of metabolic activity in vitro and in vivo (Huang *et al.*, 2002; Zipfel *et al.*, 2003; Georgakoudi and Quinn, 2012). Several endogenous molecules, including NAD(P)H are auto fluorescent. The fluorescence lifetime of NAD(P)H varies whether it is free or bound, thus can be used as a metabolic indicator (Lakowicz *et al.*, 1992). NAD(P)H in the mitochondria is known to be primarily protein bound, resulting in a fraction bound NAD(P)H that has a longer lifetime than the free NAD(P)H in the cytosol and nucleus (Blinova *et al.*, 2008; Zheng *et al.*, 2008). Thus, changes in the lifetime of NAD(P)H have been attributed to alterations in the relative levels of OXPHOS and glycolysis (Bird *et al.*, 2005; Stringari *et al.*, 2011; Quinn *et al.*, 2013). In this study, we implemented two-photon excitation of NAD(P)H and FLIM to map the temporal and spatial metabolic patterns in isolated human T cells, that is, the widely used Jurkat cell line (Abraham and Weiss, 2004) and human primary CD4^+^ T cells to follow the metabolic changes upon activation. Using metabolic inhibitors (Supplemental Figure , S7 and 8), we determined the specificity of the fraction of bound NAD(P)H measurement to defined metabolic pathways: rotenone which induces a shift from OXPHOS to glycolysis by blocking the respiratory chain increased fraction of bound NAD(P)H in cells. Besides, 2-DG, which interferes with glucose metabolism induced a decrease in fraction of bound NAD(P)H in T cells that revealed the shift toward more OXPHOS.

Our results show that T lymphocyte activation by the TCR/CD3 and the CD28 molecules rapidly (in 10 min) induces a decrease in fraction of bound NAD(P)H, witnessing a metabolic shift toward more glycolysis. The measurement of fraction of bound NAD(P)H in live cells enables an early observation of the metabolic switch upon T-cell activation while most of the studies analyze metabolic changes after days of activation (Lopes *et al.*, 2021; Russo *et al.*, 2022). The metabolic shift toward glycolysis reported in these cells can thus be due to transcription of genes encoding enzymes controlling glycolysis. Indeed, we have shown that human T lymphocytes activated by anti-CD3 and anti-CD28 activating antibodies increase transcription of genes encoding Phosphofructokinase, Glyceraldehyde-3-Phosphate Dehydrogenase, Phosphoglycerate Kinase 1, phosphoglucomutase, Enolase and Pyruvate kinase (Saitakis *et al.*, 2017), which are all enzymes controlling glycolysis. In the present study, the rapidity of the metabolic shift toward glycolysis strongly suggests that the process is transcription independent. Our results, which show a negative correlation between cell spreading and fraction of bound NAD(P)H, suggest a causal effect between actin remodeling and glycolytic activity of T cells. Indeed, direct interactions between glycolytic enzymes and filamentous actin have been reported and actin remodeling shown to regulate these enzymatic activities (reviewed in DeWane *et al.*, 2021). Such a regulation is exemplified by a study reporting that insulin induces the release of enolase A from F-actin via a phosphoinositide 3-kinase (PI3K) and Rac dependent actin remodeling. Free aldolase then leads to an increase in total aldolase activity, driving glycolytic flux in insulin-activated cells (Ung *et al.*, 2021). Interestingly, TCR/CD3 and CD28 activation also induce Rac and PI3K activation in T lymphocytes (Genot *et al.*, 1996; Reif *et al.*, 1996; Tuosto *et al.*, 1996). It would thus be interesting to explore if the rapid cytoskeleton remodeling observed in activated T cells also controls the availability of glycolytic enzymes in these cells. Alternatively, there might be no causal effect: spreading of T cells and increase in F-actin would reflect T cell activation: the more activated the cell, the more glycolytic it is.

This method was already used to show that the autofluorescence decay of NAD(P)H is sensitive to T cell subtypes and activation state (Walsh *et al.*, 2020). Heterogeneity of NAD(P)H lifetime was reported in T lymphocytes in each donor and among donors, it was also different between CD3^+^CD4^+^ and CD3^+^CD8^+^ activated T cells (Walsh *et al.*, 2020). Moreover, the fraction of free NAD(P)H was lower in quiescent T cells from the blood of healthy donors than in T cells activated for 24–72 h with tetrameric antibodies (anti-CD2/CD3/CD28) and the authors of the study reported that the lifetime of NAD(P)H was consistently the most important biomarker for differentiating quiescent and activated T cells (Walsh *et al.*, 2020). The decreased fraction of bound NAD(P)H we observed in activated T cells is consistent with this published study although the timing was different. One important difference is that we only measured the lifetime of NAD(P)H focusing on the OXPHOS and glycolysis pathways, whereas in the cited study, Walsh *et al.* (2013) measured multiple metabolic intrinsic biomarkers (lifetime of NAD(P)H, lifetime of flavin adenine dinucleotide [FAD] and optical redox ratio [defined as NAD(P)H/(NAD(P)H+FAD]) that provide complementary information about different metabolic pathways and phenotypes including fatty acid synthesis and glutaminolysis (Liu *et al.*, 2018). Moreover, we concentrated on the early metabolic changes. Using fluorescence imaging of both endogenous NAD(P)H and FAD, the authors reported an initial (at 30 min) decrease of the optical redox ratio in T lymphocytes that then increases over time (from 2 h–24 h; Walsh *et al.*, 2020). It is thus difficult to directly compare the results we report herein with the results of Walsh *et al.* (2020) due to different T-cell activation, imaging timing and biomarker measurements. Yet, it would be of high interest to characterize and compare the spatial dynamics of metabolism switch in T cells induced, at early time points, by different activators (coated, soluble crosslinked antibodies, tetramers), using multiparametric metabolic imaging with 2P-FLIM of the coenzymes NAD(P)H and FAD simultaneously (Ung *et al.*, 2021). Of note, the rapid decrease in fraction of bound NAD(P)H in activated T cells that reflects an increase in the glycolytic activity of these T lymphocytes was reported in another model. In this case, the authors used the Seahorse Extracellular Flux Analyzer, and demonstrated, in mouse T lymphocytes, a rapid (within minutes) increase of glycolysis upon activation with crosslinked anti-CD3 and anti-CD28 antibodies (Menk *et al.*, 2018). This rapid aerobic glycolysis was transcription independent and due to activation of pyruvate dehydrogenase kinase 1 (PDHK1).

Another interest of the method and work we present is that it allows a spatial visualization of the metabolism inside single cells. In our study, we show that there is a gradient of fraction of bound NAD(P)H in T lymphocytes. In nonactivated T lymphocytes, the fraction of bound NAD(P)H is evenly distributed in the cytoplasm while the central zone, representing mostly the nucleus (Supplemental Figure S9), displays the lowest values (Figure 4B). This can be explained by the absence of mitochondria in the nuclei. One interesting aspect of our results is the difference in the distribution of fraction of bound NAD(P)H in the activated cells, which shows asymmetry between the cell side in contact with the slide and its opposite. The fraction of bound NAD(P)H is higher at the contact zone than at the top of the cells, whereas in resting cells the distribution of fraction of bound NAD(P)H is symmetric at the two poles of the cells. This probably reflects the polarization of T lymphocytes toward the activating side, also called immune synapse, which induces rapid polarized transport of mitochondria at the immune synapse (Quintana *et al.*, 2007; Torralba *et al.*, 2019). It would be interesting to study if the polarity of T cells affects their metabolism. To do so, we could compare the lifetime of NAD(P)H induced by coated antibodies (as herein), which mimics the contact with an antigen-presenting cell and induce polarization and morphological changes of the T cells, with data obtained in nonpolarizing conditions (soluble antibodies).

T lymphocytes are poised to rapidly respond to diverse stimuli, in different nutrient contexts, and different physiological and pathological conditions. This ability to rapidly adapt to many situations must be accompanied by rapid changes in their cellular metabolism to fulfill their need for energy. It is thus important to develop and implement methods, which can document the metabolism changes in these immune populations. We expect that the use of 2P-FLIM of the coenzyme NAD(P)H will provide valuable information on diverse T-cell populations from nonpathological and pathological samples and in response to diverse stimulations.

## MATERIALS AND METHODS

Request a protocol through *Bio-protocol*.

### Cells

Jurkat T cells (94% homology with Jurkat clone E6.1 validated by the SSTR method on the DSMZ website) were cultured at 37°C with 5% CO_2_ in RPMI1640 Glutamax (Gibco; 61870-010) supplemented with 10% fetal calf serum (FCS; Eurobio; CVFSVF00-01) and were passed every 2–3 d at ∼0.5 × 10^6^ cells/mL.

Peripheral blood mononuclear cells (PBMCs) from healthy donors were isolated using a Ficoll density gradient. Buffy coats from healthy donors were obtained from Établissement Français du Sang in accordance with INSERM ethical guidelines. CD4^+^ T cells were purified using the total CD4^+^ negative isolation kit (Miltenyi Biotec; 130-096-533). Primary CD4^+^ T cells were activated in six-well plates coated with anti-CD3e (OKT3; 317326; BioLegend) in presence of soluble anti-CD28 (CD28.2; 302914; BioLegend) during 6 d before use and cultured in RPMI1640 Glutamax (Thermo Fisher Scientific; 61870-010) supplemented with 10% FCS (Eurobio, CVFSVF00-01), 10,000 U/mL penicillin-streptomycin (Gibco; 15140-122), 1 M Hepes (Gibco, 15630-056), 50 mM 2-Mercaptoethanol (Gibco; 31350-010) and 20 U/mL recombinant IL-2 (Immunotools; 11340025).

### Lentivirus production and Jurkat cell transduction

Replication-defective lentiviral particles were obtained by transfecting HEK-293T cells with Gag, Pol, rev, encoding plasmid (pPAX2), envelop encoding plasmid (pMD2.G) and the LifeAct-mCherry construct encoded in a pWXLD vector. After 48 h, lentiviruses were recovered in the supernatant and concentrated. Jurkat cells were transduced, and the positive fraction was sorted by flow cytometry (SH800 Cell Sorter, Sony Biotechnology) to establish a stable LifeAct-mCherry^+^ Jurkat cell line.

### T-cell activation

Poly-L-lysine coated 35-mm glass-bottom dishes (MatTek; P35GC-1.0-14-C) were coated with 0, 1µg/mL anti-CD3 (OKT3; 317326; BioLegend) and 10 µg/mL anti-CD28 (CD28.2; 302914; BioLegend) at 4°C overnight and washed with phosphate-buffered saline before use. 300,000 cells were resuspended in RPMI 1640 without phenol red (Thermo Fisher Scientific, 10363083) supplemented with 10% FCS and plated on coated dishes prior imaging.

### FLIM

Imaging was performed on a laser scanning microscope (TriMScope, Lavision Biotec, Germany). A simplified scheme of the multiphoton microscope is shown in Figure 1A. The excitation is provided by a dual-output femtosecond laser (Insight DS++, Spectra-Physics, Santa Clara, CA, USA) one with a first beam tuneable from 680 nm to 1300 nm (120 fs pulses, 80 MHz) and a second one with a fixed wavelength beam at 1040 nm (200 fs pulses). A water immersion objective (25X, NA = 1.05, XLPLN-MP, Olympus, Japan) is used to focus the laser on the sample and collect fluorescence signal. Fluorescence signal is epi-detected by a hybrid photomultiplier tube (R10467U, Hamamatsu, Japan).

To perform fluorescence lifetime microscopy of NAD(P)H, 740 nm wavelength excitation was used with a typical power of 6 mW. A blue band-pass filter was installed in front of the detector to collect NAD(P)H autofluorescence (Semrock FF01–460/80). The red fluorescence of LifeAct-mCherry is simultaneously collected with a red band-pass filter (Semrock FF01–607/70). Time-correlated single photon counting (TCSPC) electronics (Lavision Biotec, Germany) measures the arrival time of the fluorescence photons with respect to the laser pulse and reconstruct the fluorescence lifetime decay. The laser trigger is taken from the fixed wavelength beam using a photodiode (PDA10CF-EC, Thorlab). Lifetime calibration of the FLIM system was performed by measuring the lifetime of fluorescein at pH 9 with a single exponential of 4.04 ns (Figure 1C). We typically collected 200–300 photons during an acquisition time in the order of seconds for a 256 × 256 pixels image at a pixel dwell time of 334 μs/pixel. The three-dimensional (3D) FLIM imaging was performed with a Z-step of 3 µm and the total acquisition time of a 3D FLIM with 6 Z planes is in the order of two min. We verified the negligible photo perturbation induced during our longitudinal imaging protocol by using a red viability dye (IncuCyteTM Cytotox Red Reagent, Cat no. 4632) during the FLIM imaging of NAD(P)H in primary cells (Supplemental Figure S1). The red fluorescence of the viability dye is simultaneously collected with the NAD(P)H fluorescence through a red pass filter (Semrock FF01– 607/70). After 1 h, the T cells that were longitudinally imaged were still viable. In the statistical analysis of primary cells, we only considered viable cells while we excluded dead cells highlighted by the red viability dye (Supplemental Figure S1).

### T Lymphocyte imaging

T lymphocytes were imaged on a glass-bottom dish, containing a nonfluorescent assay medium (Thermo Fisher Scientific, 10363083) placed inside an incubation chamber at 37°C and 5% CO_2_ (Okolab, Pozzuoli, Italy). Imaging was performed on Jurkat T cells and primary T cells in 3D by imaging 6 Z planes with a 0.3-µm step at 10, 30, and 60 min after plating the cells on Poly-l-Lysine or Poly-l-Lysine + activating antibodies. We performed label-free fluorescence lifetime microscopy of NAD(P)H in a total of 87 region of interests (ROI) with 700 cells in two independent experiments for the stable cell line and in a total of 129 ROIs with 2463 cells in three independent experiments on three different healthy donors for the primary T cells.

To measure the metabolic trajectory in cells we treated the cells with a solution of 50 µM of rotenone (R8875; Sigma-Aldrich, St. Louis, MO, USA) to block the respiratory chain via complex I and 2-DG (D8375-10MG; Sigma-Aldrich, St. Louis, MO, USA) of 10 mM to inhibit glycolysis. Cells were preincubated with rotenone for 10 min or with 2-DG for 30 min before activation and imaging. To visualize the nucleus and mitochondria, cells were stained prior fixation with Mitotracker (Invitrogen, ref: 17501655) and the nucleus was stained after fixation and permeabilization with DAPI (Invitrogen, ref: D1306). The images were acquired with a confocal microscope, on an inverted Leica DmI8 microscope equipped with an SP8 confocal using a 63× (1.4 NA), oil immersion objective.

### Analysis of the fluorescence lifetime microscopy images

NAD(P)H intensity images were obtained using Fiji-ImageJ (NIH, Bethesda, MD, USA) as the sum of the FLIM stack. All FLIM data were processed and analyzed with a custom written MATLAB (Mathworks, Natick, MA, USA) software. FLIM data were transformed from time domain to frequency domain by using a fast Fournier transform and plotted in the phasor plot as previously described (Stringari *et al.*, 2011; Ranjit *et al.*, 2018). As our FLIM data were acquired in the time domain (Figure 1C) we transformed the multiexponential fluorescence intensity decay in every pixel of the image into a point in the two-dimensional phasor plot of coordinates *g* and *s* (Figure 1E) with the following equations:




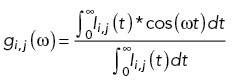

1






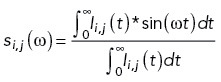

2



where *i* and *j* indicate the order pixel of the image and ω is the frequency. ω is calculated through the laser repetition rate ω = 2πf (f = 80 MHz). We calibrated our FLIM system by using a solution of fluorescein at pH 9 that has a single lifetime of 4 ns. We also measured the lifetime of free NAD(P)H in solution to be 0.4 ns as previously reported in literature (Lakowicz *et al.*, 1992). We estimated the fraction of bound NAD(P)H by calculating the distance between the experimental point 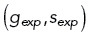 and the location of free NAD(P)H (*g*_fNADH_,*s*_fNADH_; Figure 1E) using the following equation:




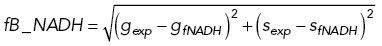

3



We note that in this calculation the location of bound NAD(P)H is not assumed and considered, because the lifetime of bound NAD(P)H is complex and highly dependent on enzymatic binding. We also note that the fraction of bound NAD(P)H represents the relative degrees of enzymatic binding, but it is not dependent on the overall concentration of NAD(P)H.

We implemented single-cell analysis to quantify the cellular mean of the fraction of bound NAD(P)H from the 3D FLIM data by using masks of single cells defined by the LifeAct-mCherry intensity for the stable cell line and by the NAD(P)H intensity for the primary T cells (Supplemental Figure S2A). First, we performed a maximum intensity Z-projection across all the Z planes of the 3D stack (Supplemental Figure S2A). Then, binary masks were created using a gaussian blur and a threshold to identify and segment each single cell (Supplemental Figure S2B) using Fiji-ImageJ (NIH, Bethesda, MD, USA). The same mask was used for every Z plane of one ROI rather than constructing separate masks for each Z plane. With a MATLAB custom written code, we also applied a threshold (5 photons) on the NAD(P)H intensity to consider only the cell signal and exclude the background at each Z plane. Then, using equation 3, we calculated the fraction of bound NAD(P)H maps (Supplemental Figure S1C) at every Z plane of the ROI by applying both the mask calculated on the maximum intensity Z-projection and the intensity threshold at every Z plane. Finally, to perform single-cell segmentation, we applied the binary masks of single cells to the three-dimensional stack fraction of bound NAD(P)H maps (Supplemental Figure S2C) and created three-dimensional metabolic maps of single cells (Supplemental Figure S2D). We then calculated the average fraction of bound NAD(P)H for every Z plane of the cell and in the entire volume of the cell considering only the pixels above the intensity threshold.

## DATA AVAILABILITY STATEMENT

The data that support the finding of the study are openly available in the Zenodo repository:

https://doi.org/10.5281/zenodo.7474053

https://doi.org/10.5281/zenodo.7473905.

## Supplementary Material





## Abbreviations used:

2-DG: 2-deoxy- d-glucose
ECAR: extracellular acidification rate
F-actin: filamentous actin
FAD: flavin adenine dinucleotide
OCR: oxygen consumption rate
OXPHOS: oxidative phosphorylation
2P: two photon
2P-FLIM: two-photon fluorescence lifetime microscopy
PBMC: peripheral blood mononuclear cells
PDHK1: pyruvate dehydrogenase kinase 1
ROI: region of interest
TCR: T cell receptor
TCSPC: time-correlated single photon counting

## References

[B1] Abdul M Sagar K Ouellette JN Cheng KP Williams JC Watters JJ Eliceiri KW (2020). Microglia activation visualization via fluorescence lifetime imaging microscopy of intrinsically fluorescent metabolic cofactors. https://doi.org/10.1117/1.NPh.7.3.035003 *7*, 035003. 10.1117/1.NPh.7.3.035003 PMC7414793 32821772

[B2] Abraham RT Weiss A (2004). Jurkat T cells and development of the T-cell receptor signalling paradigm. *Nat Rev Immunol* *4*, 301–308. 15057788 10.1038/nri1330

[B3] Alfonso-García A Smith TD Datta R Luu TU Gratton E Potma EO Liu WF (2016). Label-free identification of macrophage phenotype by fluorescence lifetime imaging microscopy. *J Biomed Opt* *21*, 046005. 27086689 10.1117/1.JBO.21.4.046005 PMC4833856

[B4] Argüello RJ Combes AJ Char R Gigan JP Baaziz AI Bousiquot E Camosseto V Samad B Tsui J Yan P, et al. (2020). SCENITH: A flow cytometry-based method to functionally profile energy metabolism with single-cell resolution. *Cell Metab* *32*, 1063–1075.e7. 33264598 10.1016/j.cmet.2020.11.007 PMC8407169

[B5] Baixauli F Martín-Cófreces NB Morlino G Carrasco YR Calabia-Linares C Veiga E Serrador JM Sánchez-Madrid F (2011). The mitochondrial fission factor dynamin-related protein 1 modulates T-cell receptor signalling at the immune synapse. *EMBO J* *30*, 1238–1250. 21326213 10.1038/emboj.2011.25 PMC3094108

[B6] Becker W (2012). Fluorescence lifetime imaging – techniques and applications. *J Microsc* *247*, 119–136. 22621335 10.1111/j.1365-2818.2012.03618.x

[B7] Bernier LP York EM Kamyabi A Choi HB Weilinger NL MacVicar BA (2020). Microglial metabolic flexibility supports immune surveillance of the brain parenchyma. *Nat Commun* *11*, 1–17. 32214088 10.1038/s41467-020-15267-z PMC7096448

[B8] Bird DK Yan L Vrotsos KM Eliceiri KW Vaughan EM Keely PJ White JG Ramanujam N (2005). Metabolic mapping of MCF10A human breast cells via multiphoton fluorescence lifetime imaging of the coenzyme NADH. *Cancer Res* *65*, 8766–8773. 16204046 10.1158/0008-5472.CAN-04-3922

[B9] Blinova K Levine RL Boja ES Griffiths GL Shi ZD Ruddy B Balaban RS (2008). Mitochondrial NADH fluorescence is enhanced by complex I binding. *Biochemistry* *47*, 9636–9645. 18702505 10.1021/bi800307y PMC3515876

[B10] Buck MD Sowell RT Kaech SM Pearce EL (2017). Metabolic instruction of immunity. *Cell* *169*, 570–586. 28475890 10.1016/j.cell.2017.04.004 PMC5648021

[B11] Bunnell SC Kapoor V Trible RP Zhang W Samelson LE (2001). Dynamic actin polymerization drives T cell receptor–induced spreading: a role for the signal transduction adaptor LAT. *Immunity* *14*, 315–329. 11290340 10.1016/s1074-7613(01)00112-1

[B12] Corrado M Pearce EL (2022). Targeting memory T cell metabolism to improve immunity. *J Clin Invest* *132*, e148546. 34981777 10.1172/JCI148546 PMC8718135

[B13] Datta R Heaster TM Sharick JT Gillette AA Skala MC (2020). Fluorescence lifetime imaging microscopy: fundamentals and advances in instrumentation, analysis, and applications. *J Biomed Opt* *25*, 1. 10.1117/1.JBO.25.7.071203 PMC7219965 32406215

[B14] DeWane G Salvi AM DeMali KA (2021). Fueling the cytoskeleton-links between cell metabolism and actin remodeling. *J Cell Sci* *134*, jcs248385. 33558441 10.1242/jcs.248385 PMC7888749

[B15] Geltink RIK Kyle RL Pearce EL (2018). Unraveling the complex interplay between T cell metabolism and function. *Annu Rev Immunol* *36*, 461. 29677474 10.1146/annurev-immunol-042617-053019 PMC6323527

[B16] Genot E Cleverley S Henning S Cantrell D (1996). Multiple p21ras effector pathways regulate nuclear factor of activated T cells. *EMBO J* *15*, 3923–3933. 8670897 PMC452103

[B17] Georgakoudi I Quinn KP (2012). Optical imaging using endogenous contrast to assess metabolic state. *Annu Rev Biomed Eng* *14*, 351–367. 22607264 10.1146/annurev-bioeng-071811-150108

[B18] He L Raddatz AD Zhou F Hwang H Kemp ML Lu H (2019). Dynamic mitochondrial migratory features associated with calcium responses during T cell antigen recognition. *J Immunol* *203*, 760–768. 31201236 10.4049/jimmunol.1800299 PMC6650333

[B19] Heaster TM Heaton AR Sondel PM Skala MC (2021). Intravital metabolic autofluorescence imaging captures macrophage heterogeneity across normal and cancerous tissue. *Front Bioeng Biotechnol* *9*, 312. 10.3389/fbioe.2021.644648 PMC8093439 33959597

[B20] Heaster TM Humayun M Yu J Beebe DJ Skala MC (2020). Autofluorescence imaging of 3D tumor-macrophage microscale cultures resolves spatial and temporal dynamics of macrophage metabolism. *Cancer Res* *80*, 5408–5423. 33093167 10.1158/0008-5472.CAN-20-0831 PMC7718391

[B21] Heikal AA (2010). Intracellular coenzymes as natural biomarkers for metabolic activities and mitochondrial anomalies. *Biomark Med* *4*, 241–263. 20406068 10.2217/bmm.10.1 PMC2905054

[B22] Huang S Heikal AA Webb WW (2002). Two-photon fluorescence spectroscopy and microscopy of NAD(P)H and flavoprotein. *Biophys J* *82*, 2811–2825. 11964266 10.1016/S0006-3495(02)75621-X PMC1302068

[B23] Irving M de Silly RV Scholten K Dilek N Coukos G (2017). Engineering chimeric antigen receptor T-cells for racing in solid tumors: Don’t forget the fuel. *Front Immunol* *8*, 267. 28421069 10.3389/fimmu.2017.00267 PMC5376574

[B24] Jang C Chen L Rabinowitz JD (2018). Metabolomics and isotope tracing. *Cell* *173*, 822–837. 29727671 10.1016/j.cell.2018.03.055 PMC6034115

[B25] Lakowicz JR Szmacinski H Nowaczyk K Johnson ML (1992). Fluorescence lifetime imaging of free and protein-bound NADH. *Proc Natl Acad Sci* USA *89*, 1271–1275. 1741380 10.1073/pnas.89.4.1271 PMC48431

[B26] Liu Z Pouli D Alonzo CA Varone A Karaliota S Quinn KP Mönger K Karalis KP Georgakoudi I (2018). Mapping metabolic changes by noninvasive, multiparametric, high-resolution imaging using endogenous contrast. *Sci Adv* *4*, eaap9302. 29536043 10.1126/sciadv.aap9302 PMC5846284

[B27] Lopes N McIntyre C Martin S Raverdeau M Sumaria N Kohlgruber AC Fiala GJ Agudelo LZ Dyck L Kane H, et al. (2021). Distinct metabolic programs established in the thymus control effector functions of γδ T cell subsets in tumor microenvironments. *Nat Immunol* *22*, 179–192. 33462452 10.1038/s41590-020-00848-3 PMC7610600

[B28] Marchingo JM Cantrell DA (2022). Protein synthesis, degradation, and energy metabolism in T cell immunity. *Cell Mol Immunol* *19*, 303. 34983947 10.1038/s41423-021-00792-8 PMC8891282

[B29] Menk AV Scharping NE Moreci RS Zeng X Guy C Salvatore S Bae H Xie J Young HA Wendell SG Delgoffe GM (2018). Early TCR signaling induces rapid aerobic glycolysis enabling distinct acute T cell effector functions. *Cell Rep* *22*, 1509–1521. 29425506 10.1016/j.celrep.2018.01.040 PMC5973810

[B30] Miskolci V Tweed KE Lasarev MR Britt EC Walsh AJ Zimmerman LJ McDougal CE Cronan MR Fan J Sauer J-D, et al. (2022). In vivo fluorescence lifetime imaging of macrophage intracellular metabolism during wound responses in zebrafish. *eLife* 11, e66080. 10.7554/eLife.66080 PMC8871371 35200139

[B31] Quinn KP Sridharan GV Hayden RS Kaplan DL Lee K Georgakoudi I (2013). Quantitative metabolic imaging using endogenous fluorescence to detect stem cell differentiation. *Sci Rep* *3*, 1–10. 10.1038/srep03432 PMC3851884 24305550

[B32] Quintana A Schwindling C Wenning AS Becherer U Rettig J Schwarz EC Hoth M (2007). T cell activation requires mitochondrial translocation to the immunological synapse. *Proc Natl Acad Sci USA* *104*, 14418–14423. 17726106 10.1073/pnas.0703126104 PMC1964825

[B33] Ranjit S Malacrida L Jameson DM Gratton E (2018). Fit-free analysis of fluorescence lifetime imaging data using the phasor approach. *Nat Protoc* *13*, 1979–2004. 30190551 10.1038/s41596-018-0026-5

[B34] Reif K Nobes CD Thomas G Hall A Cantrell DA (1996). Phosphatidylinositol 3-kinase signals activate a selective subset of Rac/Rho-dependent effector pathways. *Curr Biol* *6*, 1445–1455. 8939609 10.1016/s0960-9822(96)00749-x

[B35] Russo E Lemaître F Eatrice Corre B Chikina AS Langa-Vives F Bousso P (2022). SPICE-Met: profiling and imaging energy metabolism at the single-cell level using a fluorescent reporter mouse. *EMBO J* *41*, e111528. 35997165 10.15252/embj.2022111528 PMC9531294

[B36] Saitakis M Dogniaux S Goudot C Bufi N Asnacios S Maurin M Randriamampita C Asnacios A Hivroz C (2017). Different TCR-induced T lymphocyte responses are potentiated by stiffness with variable sensitivityDifferent TCR-induced T lymphocyte responses are potentiated by stiffness with variable sensitivity. *eLife* *6*, 1–29. 10.7554/eLife.23190 PMC5464771 28594327

[B37] Sánchez-Ramírez E Ung TPL Alarcón Del Carmen A del Toro-Ríos X Fajardo-Orduña GR Noriega LG Cortés-Morales VA Tovar AR Montesinos JJ Orozco-Solís R, et al. (2022). Coordinated metabolic transitions and gene expression by NAD+ during adipogenesis. *J Cell Biol* 221, e202111137. 10.1083/jcb.202111137 PMC9538974 36197339

[B38] Skala MC Riching KM Gendron-Fitzpatrick A Eickhoff J Eliceiri KW White JG Ramanujam N (2007). In vivo multiphoton microscopy of NADH and FAD redox states, fluorescence lifetimes, and cellular morphology in precancerous epithelia. *Proc Natl Acad Sci USA* *104*, 19494–19499. 18042710 10.1073/pnas.0708425104 PMC2148317

[B39] Smokelin IS Mizzoni C Erndt-Marino J Kaplan DL Georgakoudi I (2020). Optical changes in THP-1 macrophage metabolism in response to pro- and anti-inflammatory stimuli reported by label-free two-photon imaging. *J Biomed Opt* *25*, 014512. 31953928 10.1117/1.JBO.25.1.014512 PMC7008597

[B40] Stringari C Cinquin A Cinquin O Digman MA Donovan PJ Gratton E (2011). Phasor approach to fluorescence lifetime microscopy distinguishes different metabolic states of germ cells in a live tissue. *Proc Natl Acad Sci USA* *108*, 13582–13587. 21808026 10.1073/pnas.1108161108 PMC3158156

[B41] Stringari C Nourse JL Flanagan LA Gratton E (2012). Phasor fluorescence lifetime microscopy of free and protein-bound NADH reveals neural stem cell differentiation potential. *PLoS One* *7*, e48014. 23144844 10.1371/journal.pone.0048014 PMC3489895

[B42] Stringari C Abdeladim L Malkinson G Mahou P Solinas X Lamarre I Brizion S Galey JB Supatto W Legouis R, et al. (2017). Multicolor two-photon imaging of endogenous fluorophores in living tissues by wavelength mixing. *Sci Rep* *7*, 3792. 28630487 10.1038/s41598-017-03359-8 PMC5476668

[B43] Tantama M Martínez-François JR Mongeon R Yellen G (2013). Imaging energy status in live cells with a fluorescent biosensor of the intracellular ATP-to-ADP ratio. *Nat Commun* *4*, 2550. 24096541 10.1038/ncomms3550 PMC3852917

[B44] Torralba D Martín-Cófreces NB Sanchez-Madrid F (2019). Mechanisms of polarized cell-cell communication of T lymphocytes. *Immunol Lett* *209*, 11–20. 30954509 10.1016/j.imlet.2019.03.009

[B45] Tuosto L Michel F Acuto O (1996). p95vav associates with tyrosine-phosphorylated SLP-76 in antigen-stimulated T cells. *J Exp Med* *184*, 1161–1166. 9064333 10.1084/jem.184.3.1161 PMC2192766

[B46] Ueda H Morphew MK McIntosh JR Davis MM (2011). CD4 + T-cell synapses involve multiple distinct stages. *Proc Natl Acad Sci USA* *108*, 17099–17104. 21949383 10.1073/pnas.1113703108 PMC3193211

[B47] Ung TPL Lim S Solinas X Mahou P Chessel A Marionnet C Bornschlögl T Beaurepaire E Bernerd F Pena AM Stringari C (2021). Simultaneous NAD(P)H and FAD fluorescence lifetime microscopy of long UVA–induced metabolic stress in reconstructed human skin. *Sci Rep* *11*, 22171. 34772978 10.1038/s41598-021-00126-8 PMC8589997

[B48] Vishwasrao HD Heikal AA Kasischke KA Webb WW (2005). Conformational dependence of intracellular NADH on metabolic state revealed by associated fluorescence anisotropy. *JBiol Chem* *280*, 25119–25126. 15863500 10.1074/jbc.M502475200

[B49] Walsh AJ Cook RS Manning HC Hicks DJ Lafontant A Arteaga CL Skala MC (2013). Optical metabolic imaging identifies glycolytic levels, subtypes, and early-treatment response in breast cancer. *Cancer Res* *73*, 6164–6174. 24130112 10.1158/0008-5472.CAN-13-0527 PMC3801432

[B50] Walsh AJ Mueller KP Tweed K Jones I Walsh CM Piscopo NJ Niemi NM Pagliarini DJ Saha K Skala MC (2020). Classification of T-cell activation via autofluorescence lifetime imaging. *Nat Biomed Eng* *5*, 77–88. 32719514 10.1038/s41551-020-0592-z PMC7854821

[B51] Zheng W Li D Qu JY (2008). Time-resolved spectroscopic imaging reveals the fundamentals of cellular NADH fluorescence. *Opt Lett*, *33*, 2365–2367. 18923624 10.1364/ol.33.002365

[B52] Zipfel WR Williams RM Christiet R Nikitin AY Hyman BT Webb WW (2003). Live tissue intrinsic emission microscopy using multiphoton-excited native fluorescence and second harmonic generation. *Proc Natl Acad Sci USA* *100*, 7075–7080. 12756303 10.1073/pnas.0832308100 PMC165832

