# Supplementary material for: Label-free single-cell live imaging reveals fast metabolic switch in T lymphocytes
Source: Mol Biol Cell. 2023 Dec 14;35(1):ar11. doi: 10.1091/mbc.E23-01-0009 (PMC10881169; doi:10.1091/mbc.E23-01-0009)
Supplement: Supplementary file 1 [file mbc-35-ar11-s001.pdf]

Supplemental Materials

Molecular Biology of the Cell

Paillon *et al*.

SUPPLEMENTARY MATERIALS

List of supplementary figures:

- Figure S1: Assessing phototoxicity in primary T cells during longitudinal FLIM imaging of NAD(P)H using a viability dye.
- Figure. S2: Workflow of 3D FLIM for single cell analysis
- Figure S3: Example of single cell longitudinal analysis of the fraction bound NAD(P)H.
- Figure S4: Longitudinal analysis of the fraction of bound NAD(P)H in non-activated (Control) and activated Jurkat T cells.
- Figure S5: Metabolic shift during Jurkat T cell activation revealed by NAD(P)H FLIM.
- Figure S6: Metabolic shift during primary T cell activation revealed by NAD(P)H FLIM.
- Figure S7: Metabolic trajectory in Jurkat T cells following drug treatment.
- Figure S8: Metabolic trajectory in primary T cells following drug treatment.
- Figure S9: 3-dimensional (3D) visualization of mitochondria and nucleus in Jurkat T cells.
- Figure S10: Simultaneous analysis of primary T cell spreading and metabolic shift during activation, presented donor by donor.

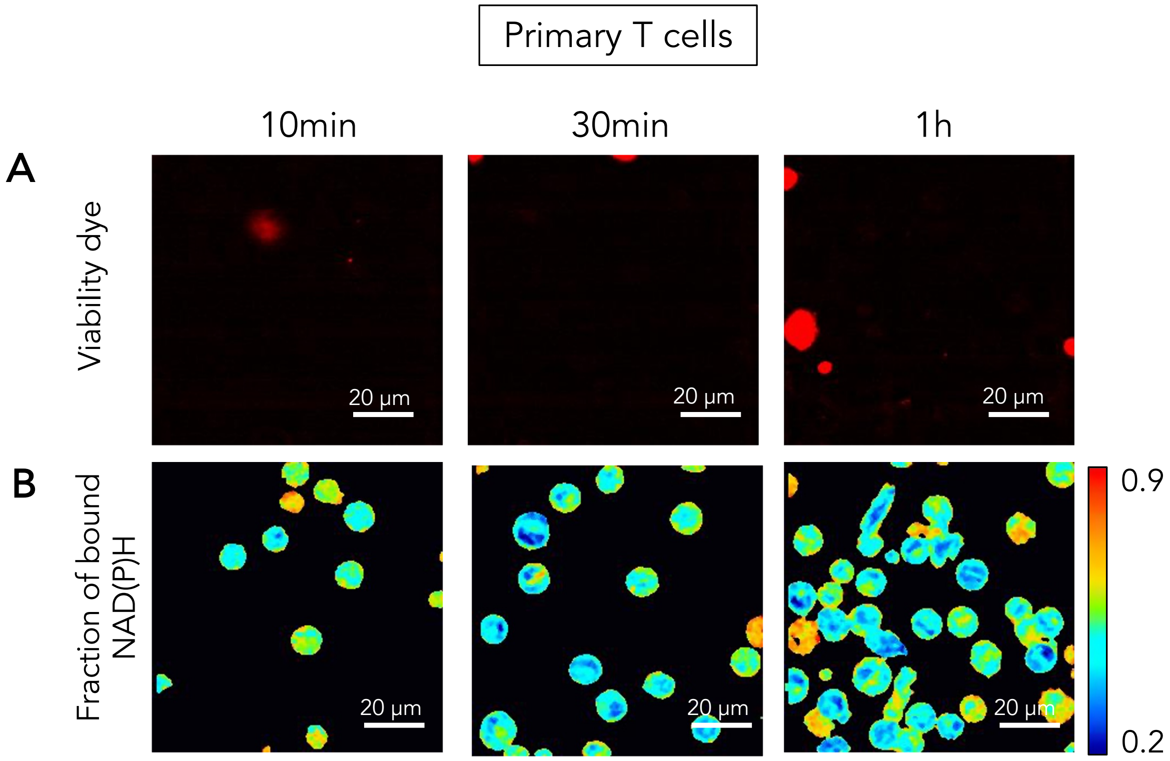

Figure S1: Assessing phototoxicity in primary T cells during longitudinal FLIM imaging of NAD(P)H using a viability dye. A. Representative images of dead cells stained with a viability dye in a ROI with primary T cells activated and imaged at 10min, 30min, and 1h during the 3D FLIM imaging protocol. After 1 hour the T cells that were longitudinally imaged were still viable. The dye was used to identify and exclude dead cells from the statistical analysis. **B.** Corresponding maps of fraction of bound NAD(P)H of the T cells from the same region of interest. We note that dead cells have a typical fraction of bound NAD(P)H that is higher than viable cells.

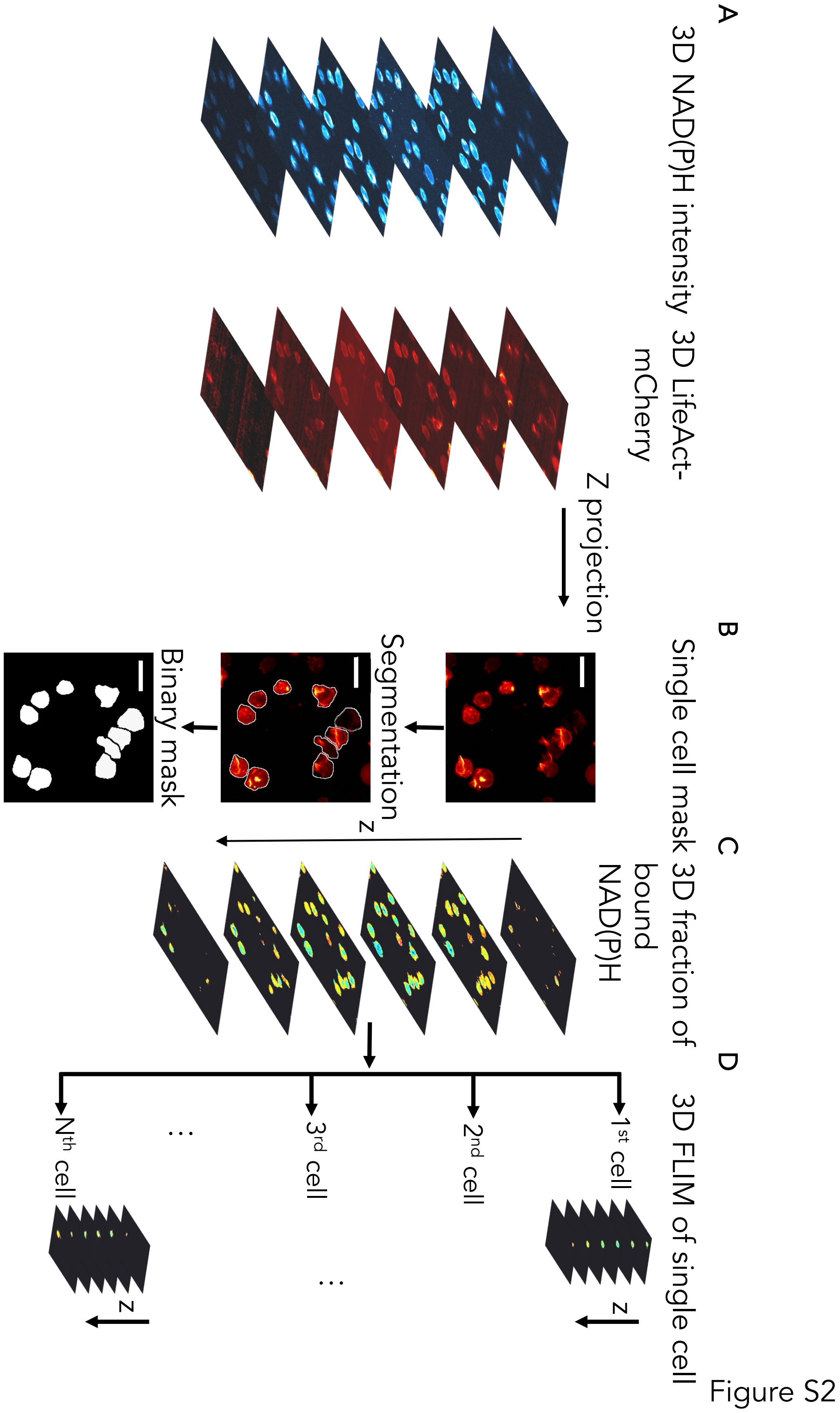

Figure S2: Workflow of 3D FLIM for single cell analysis. A. NAD(P)H FLIM and LifeAct-mCherry Z-stack images of Jurkat T cells. **B.** The Z projection is used to create single cell masks. **C.** 3D of the fraction of bound NAD(P)H. **D.** The single cell mask is applied to the 3D FLIM raw data of fraction of bound NAD(P)H to extract the FLIM data of single cell for every Z plane.

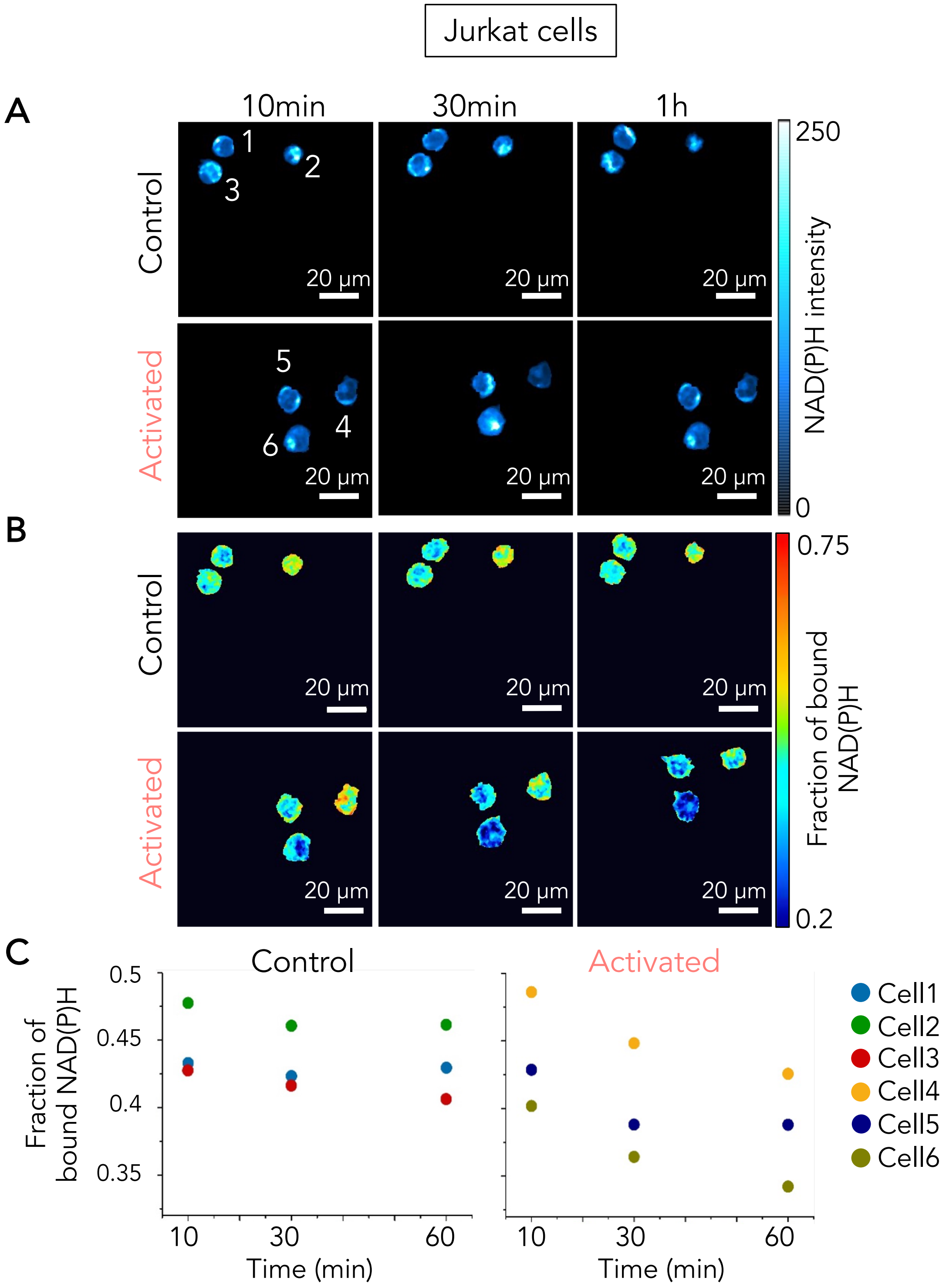

Figure S3

Figure S3: Example of single cell longitudinal analysis of the fraction bound NAD(P)H. A. Representative images of NAD(P)H intensity and **B.** maps of fraction of bound NAD(P)H of Jurkat T cells in control and activated condition at three time points (10 min, 30 min and 1 hour). **C.** Fraction of bound NAD(P)H of three representative single cells in control (Cell 1 to 3) and activated (Cell 4 to 5) conditions over time (10 min, 30 min and 1 hour).

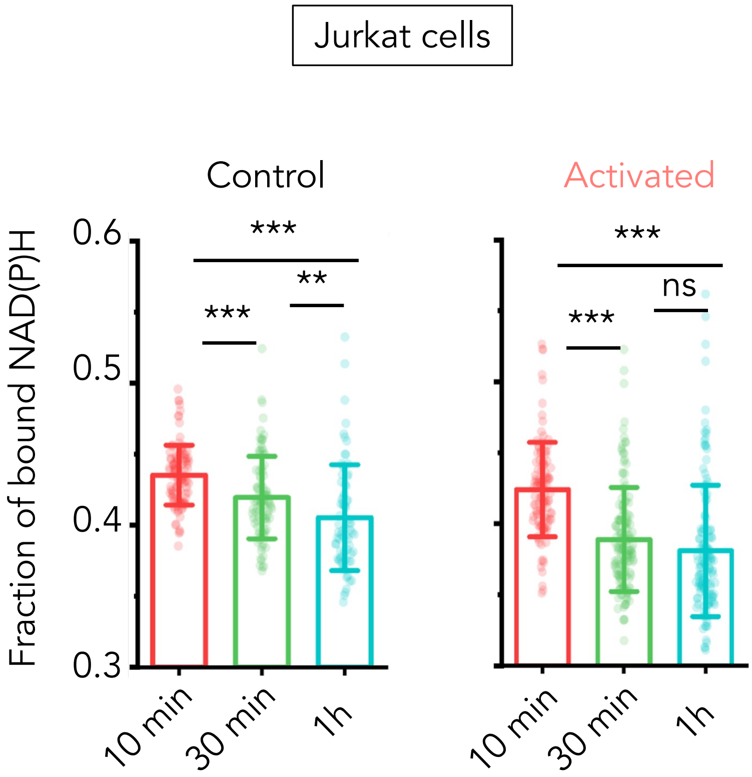

Figure S4: Longitudinal analysis of the fraction of bound NAD(P)H in non-activated (Control) and activated Jurkat T cells. A. Quantification of fraction of bound NAD(P)H of Jurkat T cells over time (10 min, 30 min and 1 hour) in control and activated condition. Each dot represents one cell. Mean with standard deviation range are shown. T test:**P ≤ 0.01, ***P<0.001. N= 2 experiments.

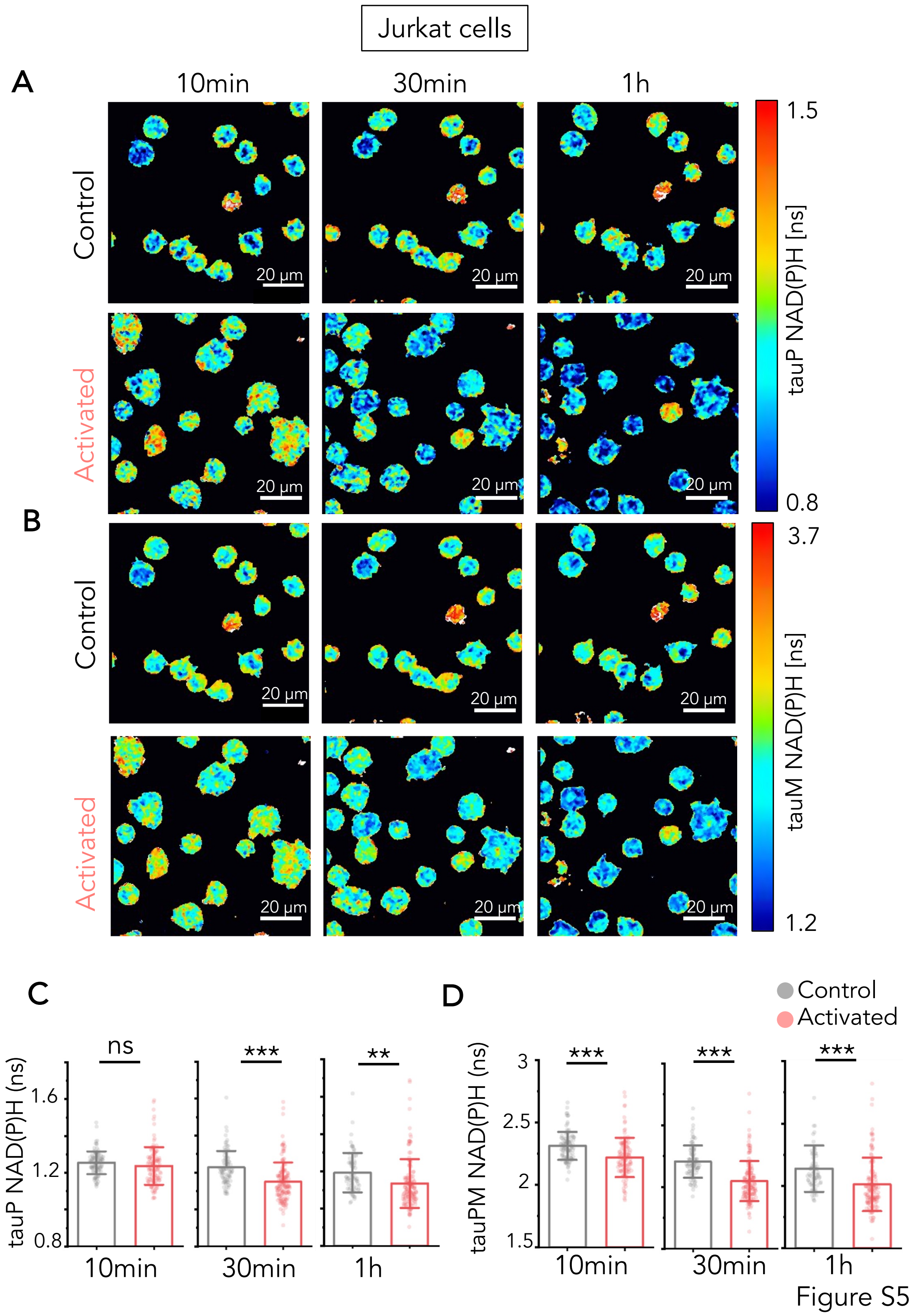

Figure S5: Metabolic shift during Jurkat T cell activation revealed by NAD(P)H FLIM. A. Representative images of phase lifetime (tauP) and **B.** modulation lifetime (tauM) of NAD(P)H of Jurkat T cells in control and activated condition at three time points (10 min, 30 min and 1 hour). Quantification of tauP (**C**) and tauM (**D**) of NAD(P)H over time of control and activated Jurkat T cells (10 min, 30 min and 1 hour). Each dot is one cell, data are presented by mean with standard deviation range. T-test: $^{**}P \leq 0.01$, $^{***}P<0.001$.

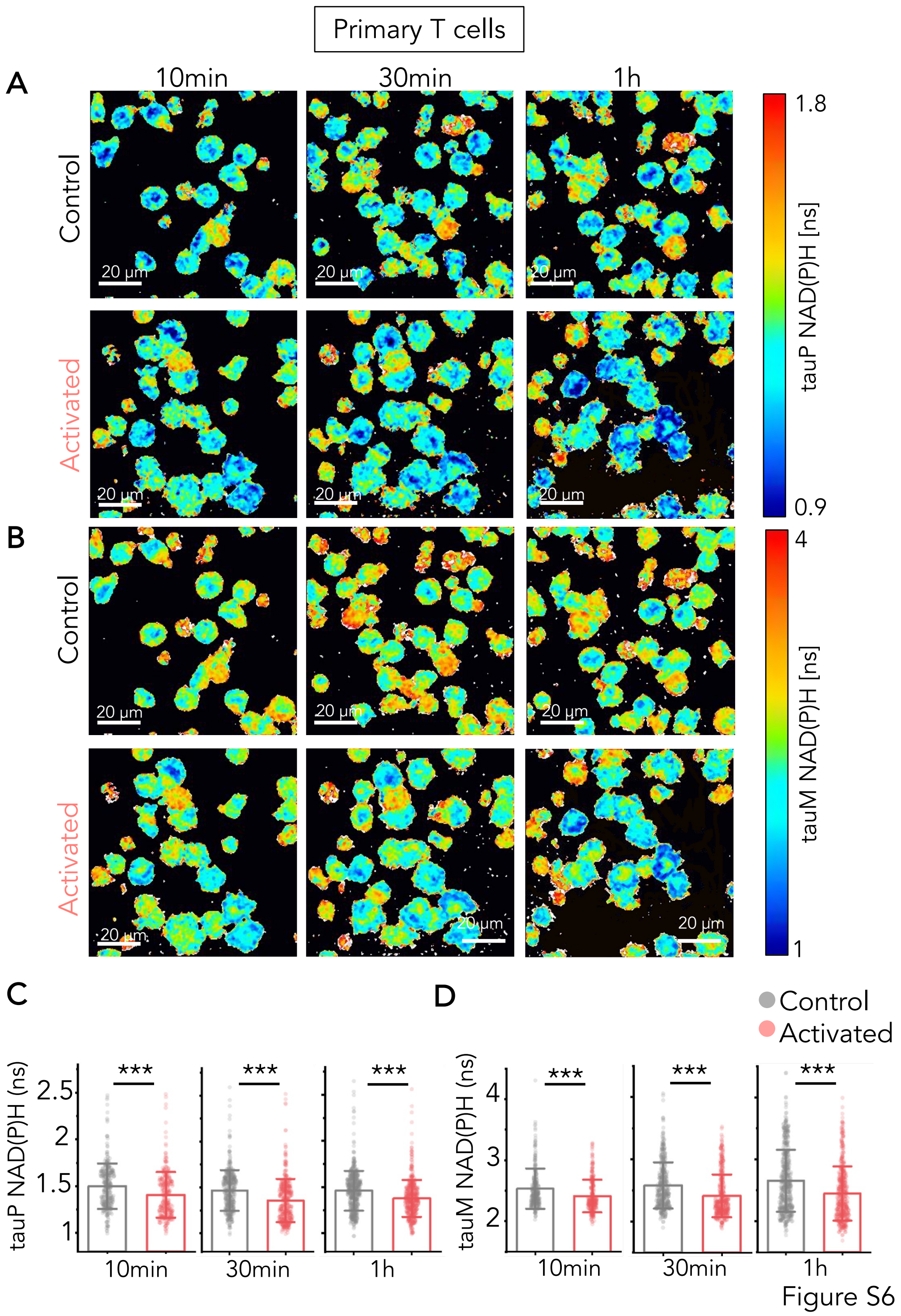

Figure S6: Metabolic shift during primary T cell activation revealed by NAD(P)H FLIM. A. Representative images of phase lifetime (tauP) and **B.** modulation lifetime (tauM) of NAD(P)H of primary T cells in control and activated condition at three time points (10 min, 30 min and 1 hour). Quantification of tauP (**C**) and tauM (**D**) of NAD(P)H over time of control and activated Jurkat T cells (10 min, 30 min and 1 hour). Data are presented by mean with standard deviation range. T test: ***P<0.001.N = 3 independent experiments with 3 different healthy donors. Data presented are means for single cells from the 3 donors.

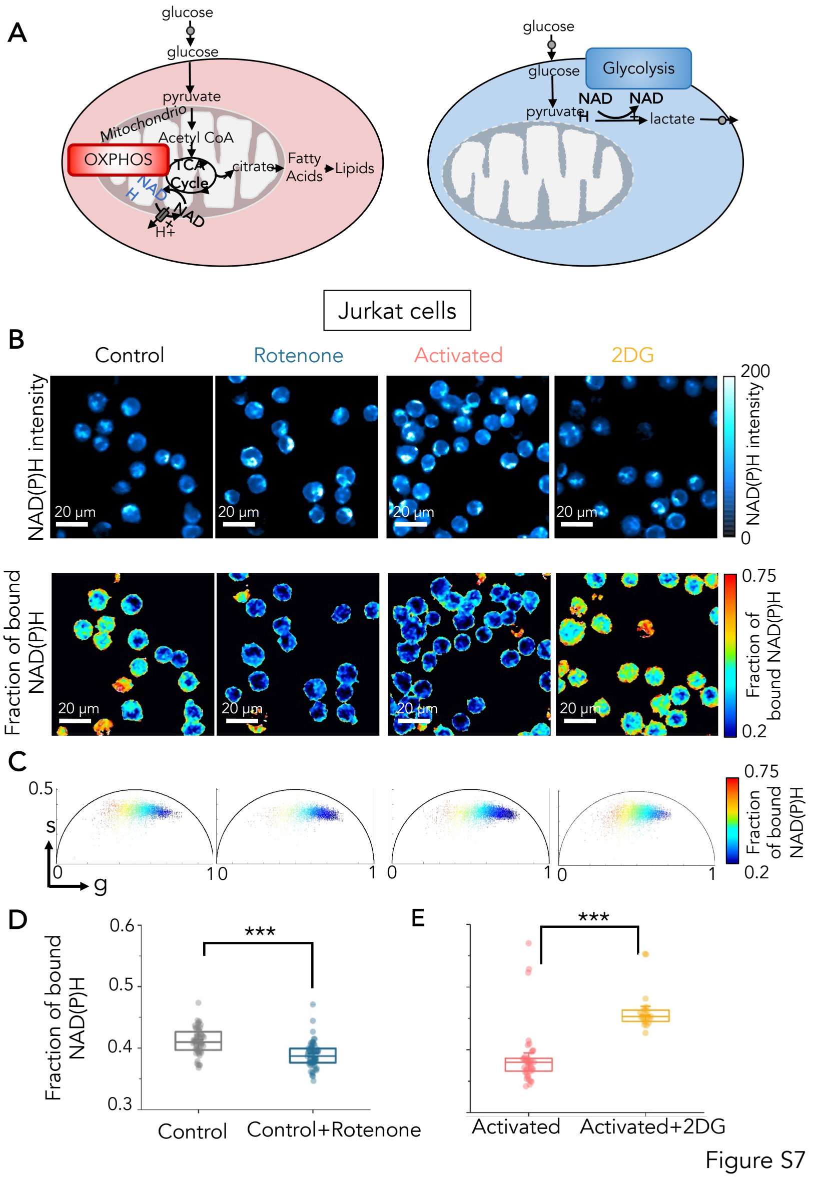

Figure S7: Metabolic trajectory in Jurkat T cells following drug treatment. A. Schematic representation of the metabolic pathways inside the cells: oxidative phosphorylation (OXPHOS) and glycolysis. Glucose breakdown through glycolysis and the TCA (tricarboxylic acid) cycle generates reduced NADH and $FADH_2$. Cells, with a glycolytic phenotype, rapidly generate ATP in the cytoplasm and are characterized by a high ratio of NADH/NAD^+ and a low fraction of bound NAD(P)H. Cells relying on oxidative phosphorylation convert glucose to pyruvate, which is then oxidized in the TCA cycle generating the majority of ATP in the mitochondria and are characterized by a low ratio of NADH/NAD^+ and a high fraction of bound NAD(P)H. **B.** Representative intensity images (top panel) and fraction of bound NAD(P)H maps (bottom panel) of non-activated (control) Jurkat T cells pretreated or not with 50 µM rotenone for 10 minutes (2 left raws) and of activated Jurkat T cells pretreated or not with 10mM of 2-Deoxy-D-glucose (2DG). Rotenone blocks the respiratory chain and leads to a decrease fraction of bound NAD(P)H, while 2-Deoxy-D-glucose prevents glycolysis and leads to an increase of bound NAD(P)H. **C.** Representative phasor plot of Jurkat T cells in different conditions: control, control treated with rotenone, activated, activated treated with 2-Deoxy-D-glucose. **D.** Decrease of fraction of bound NAD(P)H after rotenone treatment in control. **E.** Increase of fraction of bound NAD(P)H in activated Jurkat T cells with 2-Deoxy-D-glucose. Data are presented by mean with standard deviation range. T test: *** $P \leq 0.001$.

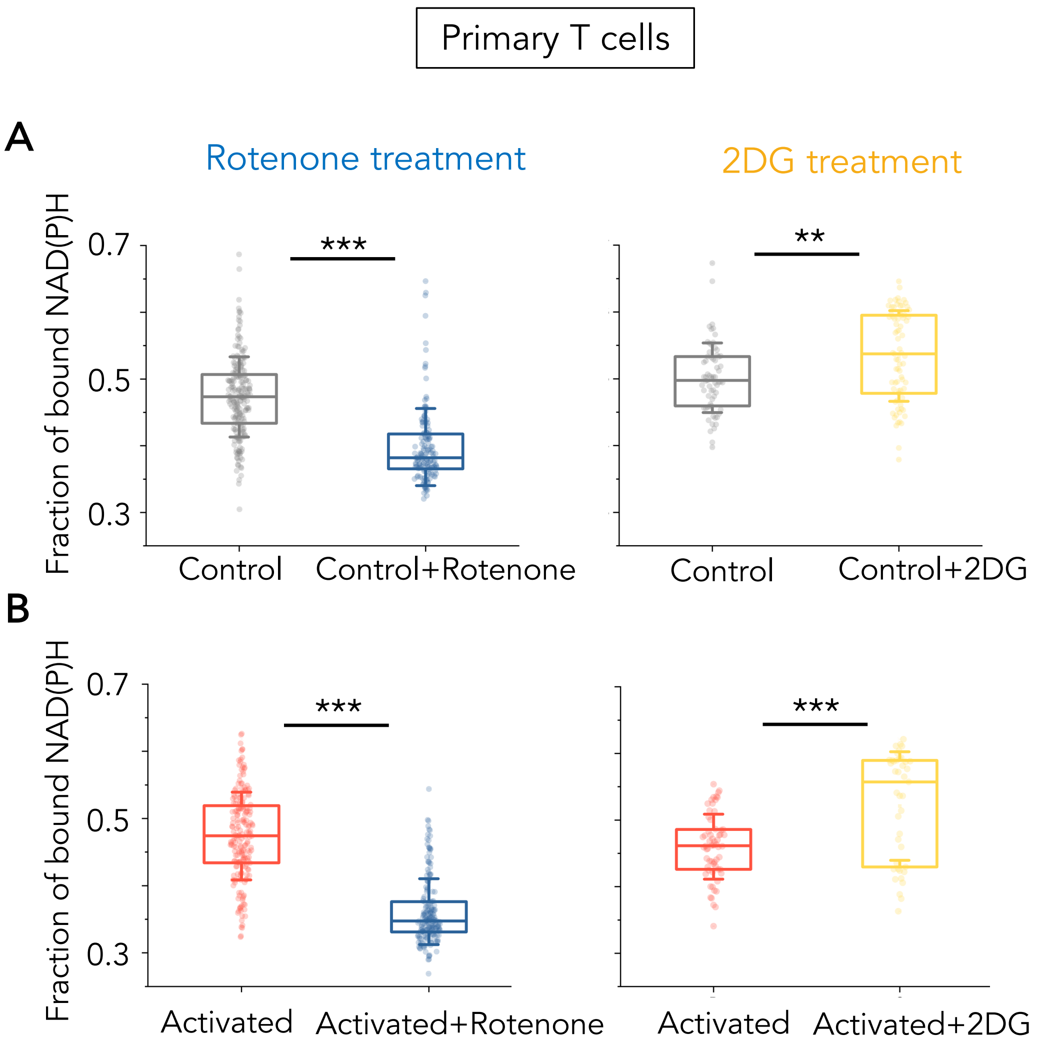

Figure S8: Metabolic trajectory in primary T cells following drug treatment. A. Quantification of fraction of bound NAD(P)H in primary T cells after rotenone (50 µM, 10 minutes) treatment in T cells from donor 2 or 2DG (10 mM, 30 minutes) treatment in T cells from donor 3 in control (non-activated) condition. **B.** Quantification of fraction of bound NAD(P)H in primary T cells after rotenone (50 µM, 10 minutes) treatment in T cells from donor 2 or 2DG (10 mM, 30 minutes) treatment in T cells from donor 3 in activated condition. Data are presented by mean with standard deviation range. T test: **$P \leq 0.01$, *** $P \leq 0.001$.

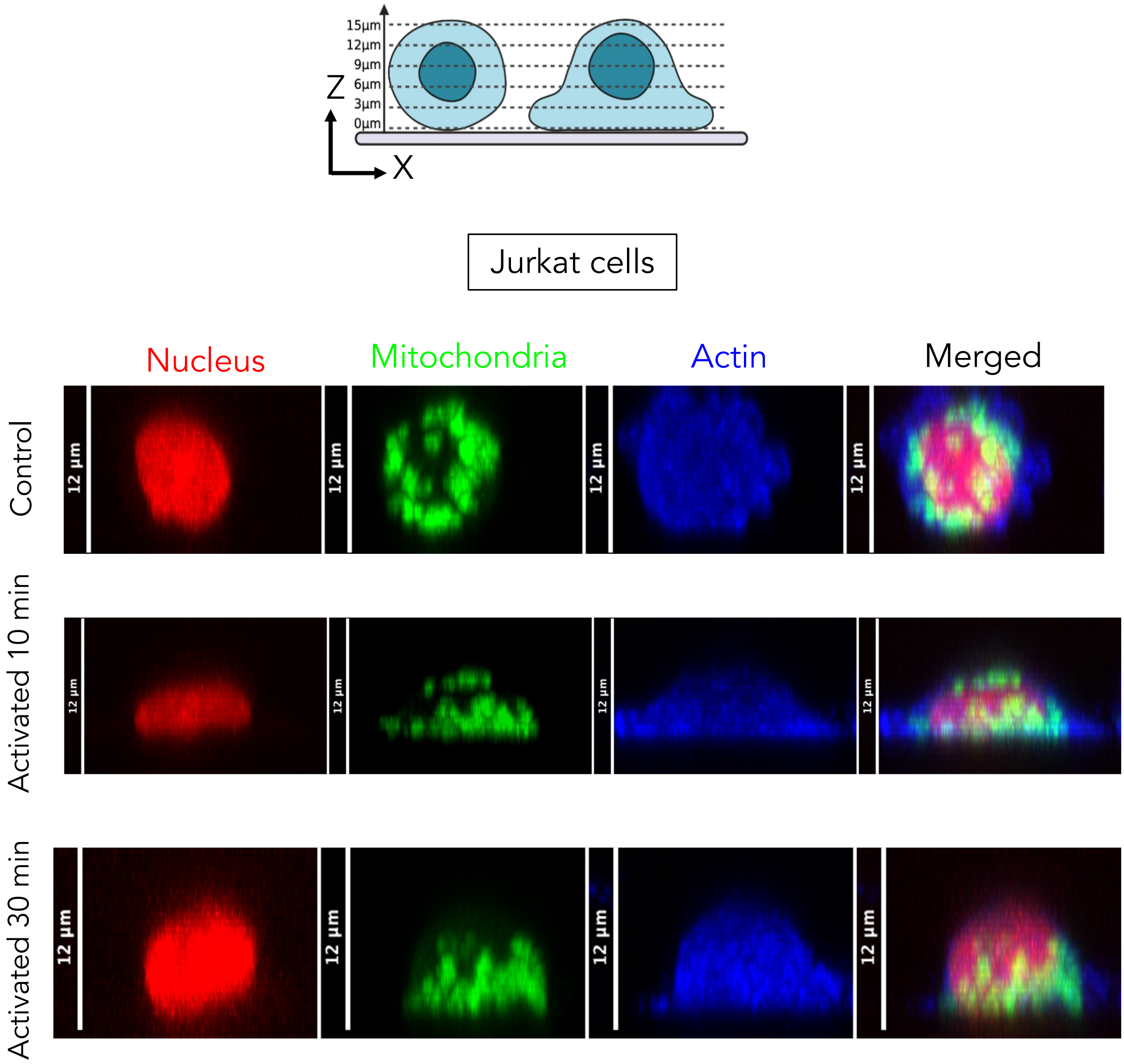

Figure S9: 3-dimensional (3D) visualization of mitochondria and nucleus in Jurkat T cells. Schematic presentation of the 3D imaging of Jurkat T cells and representative images of Jurkat T cells spreading on glass surfaces coated with poly-L-lysine (control) or poly-L-lysine and anti-CD3/CD28 antibodies (activated) for 10 min or 30 min. The F-actin was visualized with LifeAct-mCherry, mitochondria were stained prior fixation with Mitotracker and the nucleus was stained after fixation and permeabilization with DAPI. Images were acquired with a confocal microscope and Z stacks were reconstituted to visualize mitochondria and nucleus distribution across the cell in each condition.

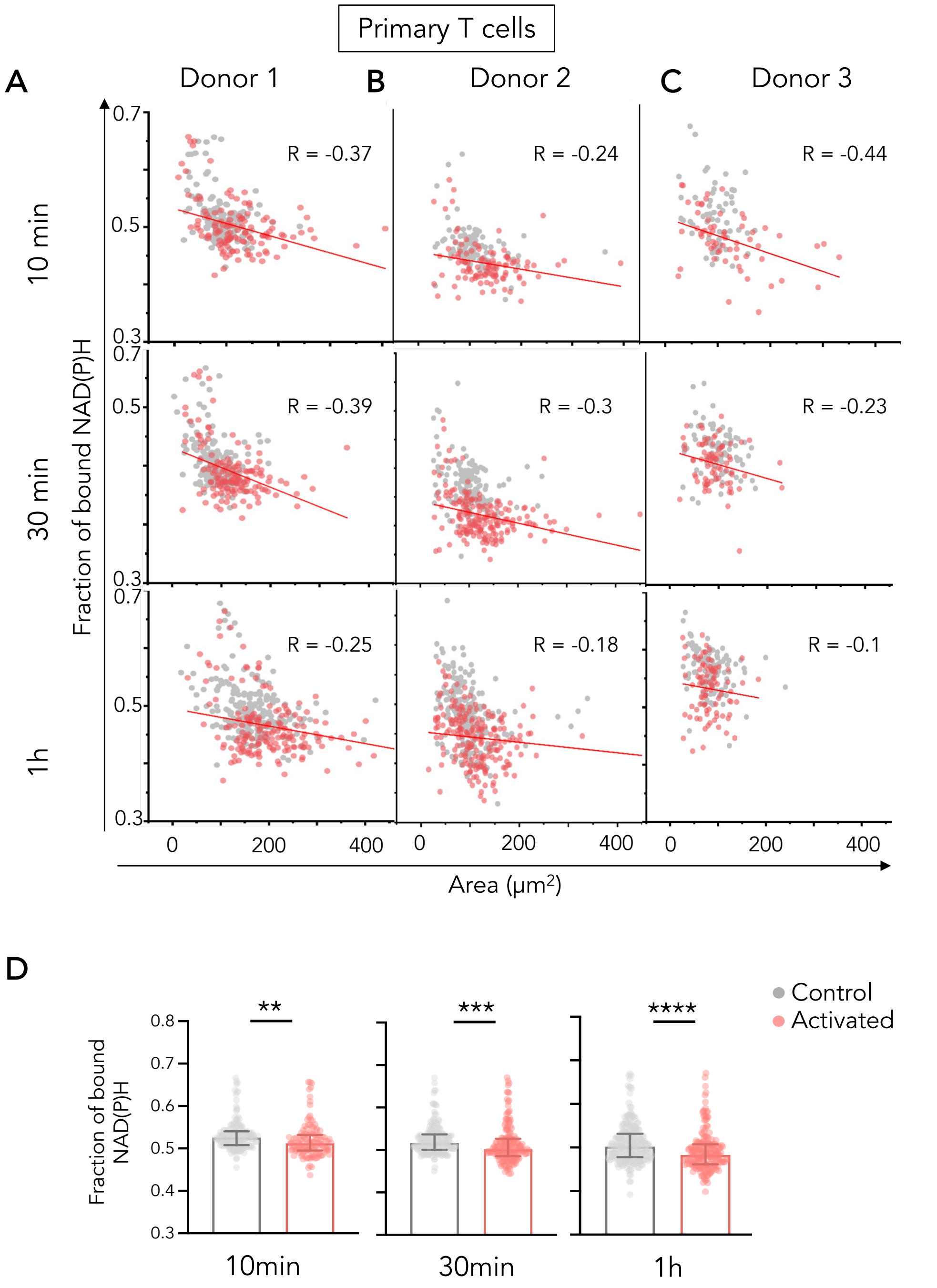

Figure S10

Figure S10: Simultaneous analysis of Primary T cell spreading and metabolic shift during activation, presented donor by donor. A-C. Correlation plots between cell areas and fraction of bound NAD(P)H of control and activated primary T cells over time (10 min, 30 min and 1 hour) for three donors. **D.** Representative quantification of the fraction of bound NAD(P)H over time (10 min, 30 min and 1 hour) in one donor (Donor 1 from figure 3). Data are presented by mean with standard deviation range. T test: **$P \leq 0.01$, ***$P<0.001$, ****$P<0.0001$.
