# Supplementary material for: Label-free single-cell live imaging reveals fast metabolic switch in T lymphocytes
Source: Mol Biol Cell. 2023 Dec 14;35(1):ar11. doi: 10.1091/mbc.E23-01-0009 (PMC10881169; doi:10.1091/mbc.E23-01-0009)
Supplement: Supplementary file 2 [file mbc-35-ar11-s002.pdf]

Label-free single-cell live imaging reveals fast metabolic switch in T lymphocytes

Noémie Paillon, Ung Thi Phuong Lien, Stephanie Dogniaux, Chiara Stringari, and Claire Hivroz

Corresponding author(s): Claire Hivroz, Institut Curie

Review Timeline:

Submission Date:	2023-01-12
Editorial Decision:	2023-03-23
Revision Received:	2023-09-29
Accepted:	2023-10-27

Editor-in-Chief: Matthew Welch

Transaction Report:

(Note: With the exception of the correction of typographical or spelling errors that could be a source of ambiguity, letters and reports are not edited. The original formatting of letters and referee reports may not be reflected in this compilation.)

RE: Manuscript #E23-01-0009
TITLE: Label-free single-cell live imaging reveals fast metabolic switch in T lymphocytes

Dear Dr. Hivroz:

The authors should pay particular attention to the reviewer's concerns around the health of the cells following exposures to the 12mW power. In addition, while the CD4+ Jurkat cell model is a useful model to develop the approach, it is a T cell lymphoma and may have very different metabolic behaviors. It is therefore critical to examine whether primary CD4+ T cells behave the same way as Jurkat T cells. The authors should therefore increase the n for the experiments examining primary human T cells, and include this critical data in the body of the manuscript. The authors should also explicitly include the number of experiments reported in the figures, as well as a power analysis to ensure that the n is appropriate to make statistical comparisons.

Sincerely,

Avery August
Monitoring Editor
Molecular Biology of the Cell

Dear Dr. Hivroz,

The review of your manuscript, referenced above, is now complete. The Monitoring Editor has decided that your manuscript is not acceptable for publication at this time, but may be deemed acceptable after specific revisions are made, as described in the Monitoring Editor's decision letter above and the reviewer comments below.

A reminder: Please do not contact the Monitoring Editor directly regarding your manuscript. If you have any questions regarding the review process or the decision, please contact the MBoC Editorial Office (mboc@ascb.org).

When submitting your revision include a rebuttal letter that details, point-by-point, how the Monitoring Editor's and reviewers' comments have been addressed. (The file type for this letter must be "rebuttal letter"; do not include your response to the Monitoring Editor and reviewers in a "cover letter.") Please bear in mind that your rebuttal letter will be published with your paper if it is accepted, unless you haveopted out of publishing the review history.

Authors are allowed 90 days to submit a revision. If this time period is inadequate, please contact us at mboc@ascb.org.

Revised manuscripts are assigned to the original Monitoring Editor whenever possible. However, special circumstances may preclude this. Also, revised manuscripts are often sent out for re-review, usually to the original reviewers when possible. The Monitoring Editor may solicit additional reviews if it is deemed necessary to render a completely informed decision.

In preparing your revised manuscript, please follow the instruction in the Information for Authors (www.molbiolcell.org/info-for-authors). In particular, to prepare for the possible acceptance of your revised manuscript, submit final, publication-quality figures with your revision as described.

To submit the rebuttal letter, revised manuscript, and figures, use this link: Link Not Available

Please contact us with any questions at mboc@ascb.org.

Thank you for submitting your manuscript to Molecular Biology of the Cell. We look forward to receiving your revised paper.

Sincerely,

Eric Baker
Journal Production Manager
MBoC Editorial Office
mbc@ascb.org

Reviewer #1 (Remarks to the Author):

Major:
1. Authors have referred to the intrinsic coenzyme as NADH. However, it is known that NADPH has similar spectral and lifetime properties. This issue is usually dealt with by the field by denoting it as NAD(P)H. Authors should consider adopting it to be consistent with the field.
2. Similar 'fb NADH' is a new abbreviation and might cause confusion. Usually its denoted as bound fraction.
3. Why did the authors used the CD4+subtype and and jurkat T cells for this study?
4. 12mW power at the sample seems too high. Consider assessing health of the cells after such exposures.
5. For the T Lymphocytes imaging, it is mentioned that for primary cells only 1 experiment was performed. Does this mean the cells were obtained from 1 donor? This seems like a small number and might not be representative. Consider including alteast 3 donors.
6. The authors should consider plotting the lifetime data too along with the bound fraction. This would make the results comparable to other studies/methods of lifetime analysis.
7. Explain what kind of z projection was done (Fig S1A)- summations/ maximum intensity..? Also, why was this chosen over creating separate mask for each z-plane
8. Explain the steps for segmentation (eg was any thresholding performed?) for reproducibility.
9. Consider plotting the variables of equation 3 on a phasor plot for easier understanding
10. Fig 1B requires further explanation that what's provided in the legend else the authors could cite previous articles explaining the principle instead of including the diagram
11. Authors mentioned that they did not observe any photodamage after longitudinal imaging. How was photodamage evaluated? Consider including the data.
12. Authors should consider adding activation markers to their experiment to selectively identify cells that have been activated, for examples...
13. For the rotenone and 2DG experiments, author should include these controls: activated t cells+ rotenone and unstimulated +2DG
14. For the 2DG experiment, were the cells preincubated with 2DG before activation? Please include details of these experiments in methods
15. In the section, 'Monitoring fraction of bound NADH in single Jurkat...', authors mentions heterogeneity in previous paragraph but no heterogeneity analysis was performed.
16. For 3D analysis, please plot the NADH intensity profile vs z to confirm that the change is not driven by lack of photons. A zero cross correlation between the two would be a convincing affirmation .
17. To confirm that the authors are imaging mitochondria and nucleus at the depths that they are imaging, it would be convincing to image the cells with fluorescent markers for nucleus and mitochondria and perform a depth comparison
18. For the LifeAct-mCherry experiment, could the authors perform a time lapse analysis mCherry signal from 0min to 10 or more to show significant increase at 10 mins?

Minor:
1. In the abstract mention the subtype of T cells used in this study
2. Introduction, second paragraph, 2nd line'...we can cite..., and 4th line ' we can also cite..' please consider rephrasing this.
3. Section: analysis of images, 1st line in 1st paragraph, '..treated with Fiji-ImageJ.." what does that mean?
4. The title of the 2nd results ..'upon time' does not make sense
5. Ref 12 and 24 are the same.

Answers to reviewers

Reviewer #1:

Major:

1. Authors have referred to the intrinsic coenzyme as NADH. However, it is known that NADPH has similar spectral and lifetime properties. This issue is usually dealt with by the field by denoting it as NAD(P)H. Authors should consider adopting it to be consistent with the field.
Answer: Since the measured fluorescence may include both NADH and NADPH, we agree with the reviewer to use NAD(P)H when talking about experimentally measured autofluorescence signal. We updated the change in the main text and figures.

2. Similar 'fb NADH' is a new abbreviation and might cause confusion. Usually its denoted as bound fraction.
Answer: We used "Fraction of bound NAD(P)H" instead of 'fb NADH in the main text and all the figures. We note that we estimated the fraction of bound NAD(P)H by simply calculating the distance between the experimental point and the location of free NAD(P)H. In this calculation the location of bound NAD(P)H is not assumed and considered, because the lifetime of bound NAD(P)H is complex and highly dependent on enzymatic binding. We also note that the fraction of bound NAD(P)H represents the relative degrees of enzymatic binding, but it is not dependent on the overall concentration of NAD(P)H. We added some sentences in the materials and methods section (page 8) and in the results section (page 9) of the main text and in the caption of Fig 1 to better clarify these concepts.

3. Why did the authors used the CD4+ subtype and Jurkat T cells for this study?
Answer: We used the Jurkat T cells because this is a model cell line that is widely used by immunologists studying T cell activation (stated in the main text). For the primary T cells, we used $CD4^+$ helper T cells because Jurkat cells are also $CD4^+$.

4. 12mW power at the sample seems too high. Consider assessing health of the cells after such exposures.
Answer: In this paper we implemented a FLIM imaging protocol starting from the methods used in a previous study of our group (*1*). In this previous paper, we used a 12 mW power because we performed a single-shot imaging of cells in culture, thus the cells were not impacted by this power. In the present study, we optimized the imaging and the FLIM illumination conditions to perform longitudinal studies of the same cells over 1 hour, therefore we used a lower power of ~6 mW to perform 3D imaging overtime. We corrected this value in the material and methods section, since we reported by mistake the power value of the previous paper.
Both in the materials and methods (page 6,7) and in the results section (page 9) we explained how we determined the minimal illumination conditions permitting longitudinal 2P-FLIM of NAD(P)H with a good signal to noise ratio and with negligible photo-perturbation.

We assessed the health of the cells with our imaging conditions by using a red viability dye (IncuCyteTM Cytotox Red Reagent, Cat no. 4632) during the FLIM imaging of NAD(P)H in primary cells (Fig S1).
We showed that after 1 hour the T cells that were longitudinally imaged were still viable:

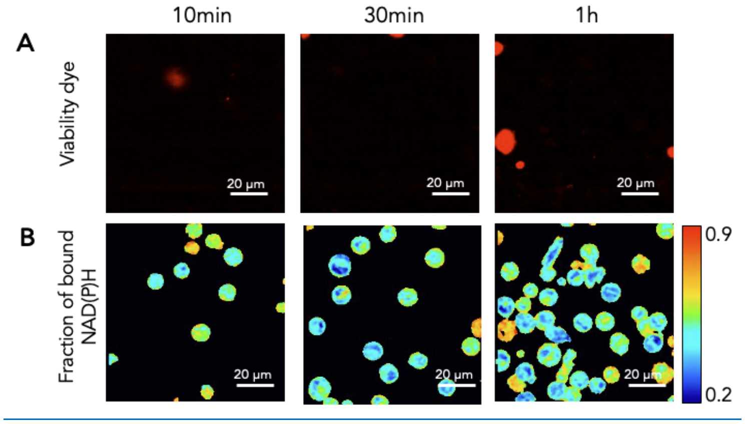

Figure S1: Assessing phototoxicity in primary T cells during longitudinal FLIM imaging of NAD(P)H using a viability dye. A. Representative images of dead cells stained with a viability dye in a ROI with primary T cells activated and imaged at 10min, 30min, and 1h during the 3D FLIM imaging protocol. After 1 hour the T cells that were longitudinally imaged were still viable. The dye was used to identify and exclude dead cells from the statistical analysis. **B.** Corresponding maps of fraction of bound NAD(P)H of the T cells from the same region of interest. We note that dead cells have a typical fraction of bound NAD(P)H that is higher than viable cells.

5. For the T Lymphocytes imaging, it is mentioned that for primary cells only 1 experiment was performed. Does this mean the cells were obtained from 1 donor? This seems like a small number and might not be representative. Consider including at least 3 donors.
Answer: We performed two additional experiments with T cells from healthy donors. The data on primary cells are now presented with 3 donors (see figures 3, 6, S6, S10) plus metabolic trajectory with drug treatment (figure S8).

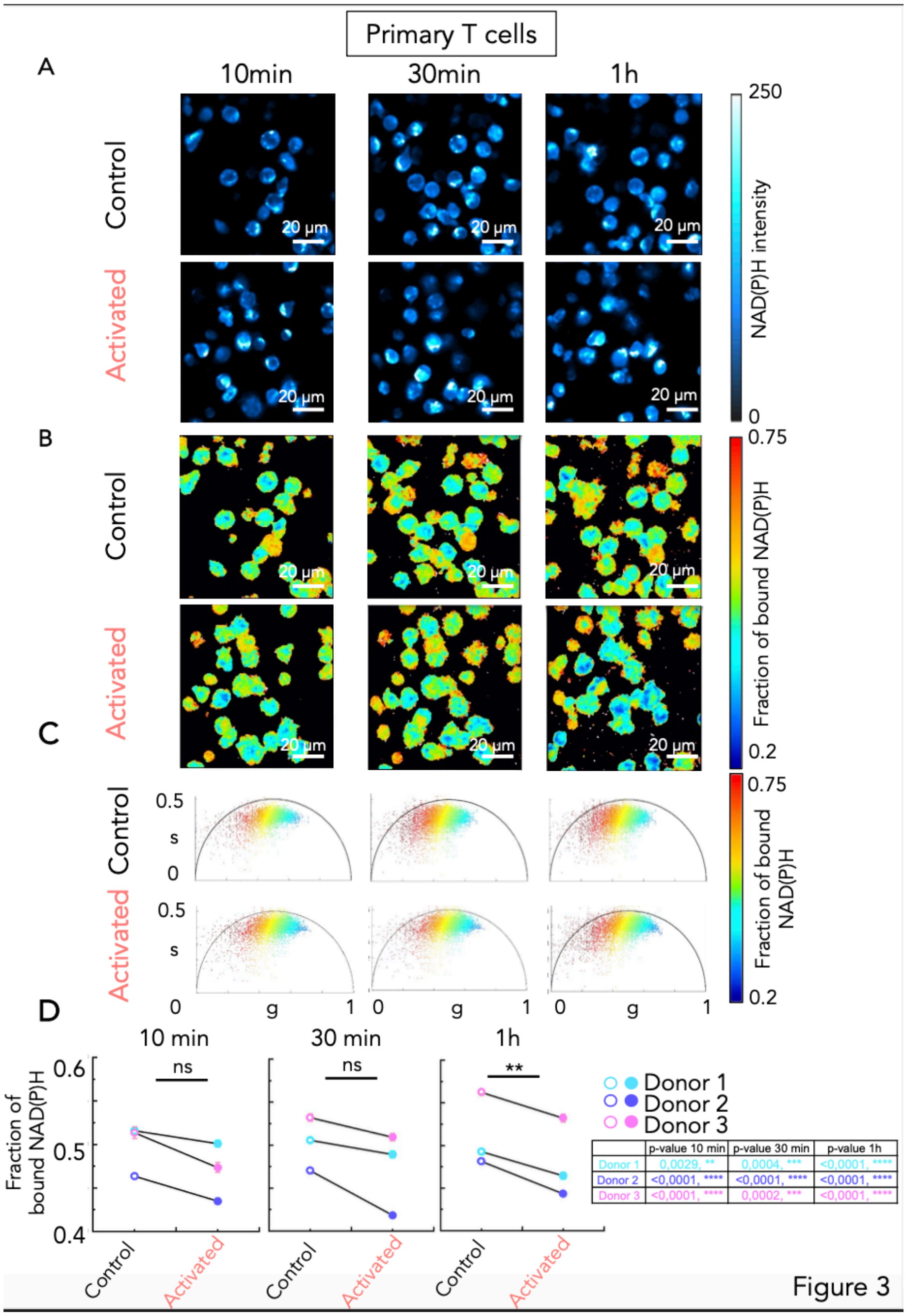

Figure 3: Metabolic shift during primary T cell activation revealed by NAD(P)H FLIM. A-D. Human primary CD4+ T cells were incubated on glass surfaces coated with poly-L-lysine alone (control), or poly-L-lysine with anti-CD3/CD28 antibodies (activated). For each condition, 3 different regions of interest were acquired at three time points (10 min, 30 min and 1 hour). **A.** Representative images of NAD(P)H intensity, **B.** maps of fraction of bound NAD(P)H and **C.** phasor plots of primary T cells. Each point in the phasor plot is color coded following the fraction of bound NAD(P)H. **D.** Fraction of bound NAD(P)H measured in primary T cells in control or activated condition at the different time points. N = 3 independent experiments with 3 different healthy donors. Data presented on the left panel are means for each donor, data presented in the table are the p-values from the paired T test done on each donor between control and activated condition. T test, **P≤ 0.01.

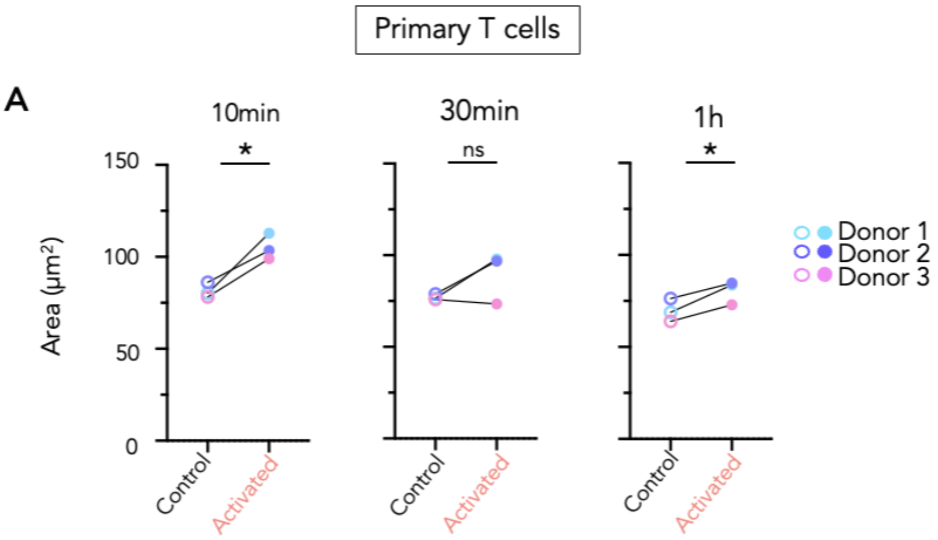

	p-value 10 min	p-value 30 min	p-value 1h
Donor 1	<0,0001, ****	<0,0001, ****	<0,0001, ****
Donor 2	0,0019, **	<0,0001, ****	0,0325, *
Donor 3	0,0036, **	0,5717, ns	0,0271, *

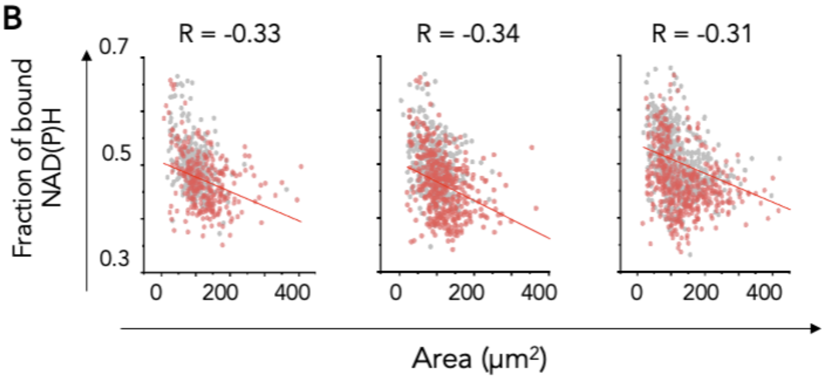

Fig. 6: Simultaneous analysis of Primary T cell spreading and metabolic shift during activation. The primary cells with 3 donors were incubated on glass surfaces coated with poly-L-lysine alone (control), or poly-L-lysine with anti-CD3/CD28 antibodies (activated) and images of 3 different regions of interest were acquired at the following time points: 10 min, 30 min and 1 hour. N = 3 independent experiments with 3 different healthy donors. **A.** Quantification of primary T cell area over time (10 min, 30 min and 1 hour) in control and activated T cells. Area of each cell was determined on the NAD(P)H intensity channel, by making a binary mask defining each cell's size. Data is presented by median with interquartile range, with each dot representing one measured cell of 3 donors. Data presented in the table are the p-values from the paired T test done on each donor between control and activated condition. T test: * $P \leq 0.05$. **B.** Correlation plot between the measured areas and fraction of bound NAD(P)H of control (grey dots) and activated (red dots) T cells over time (10 min, 30 min and 1 hour). Every point represents the mean of single cells from the 3 donors.

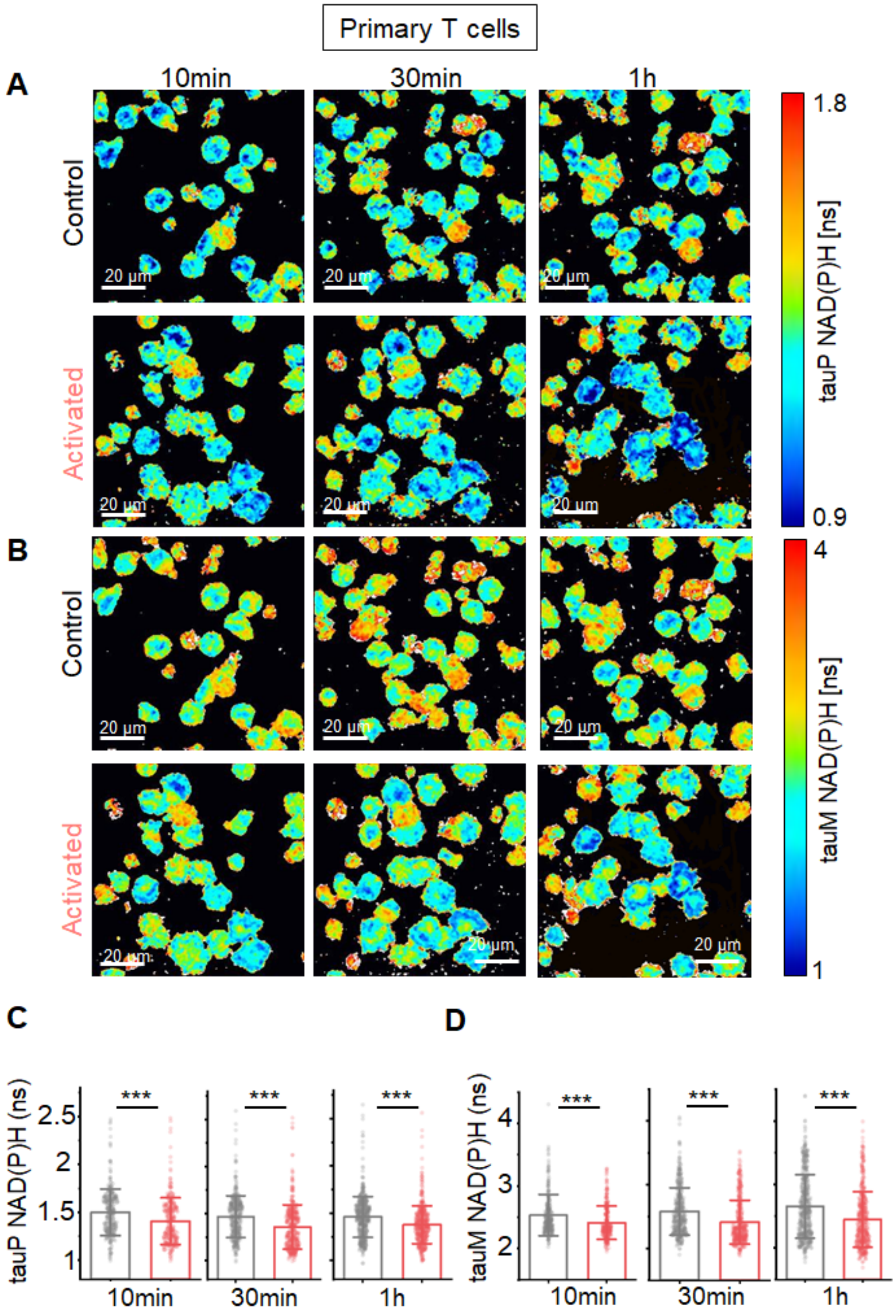

Figure S6: Metabolic shift during primary T cell activation revealed by NAD(P)H FLIM. A. Representative images of phase lifetime (tauP) and **B.** modulation lifetime (tauM) of NAD(P)H of primary T cells in control and activated condition at three time points (10 min, 30 min and 1 hour). Quantification of tauP (**C**) and tauM (**D**) of NAD(P)H over time of control and activated Jurkat T cells (10 min, 30 min and 1 hour). Data are presented by mean with standard deviation range. T test: ***P<0.001.N = 3 independent experiments with 3 different healthy donors. Data presented are means for single cells from the 3 donors. N = 3 independent experiments with 3 different healthy donors.

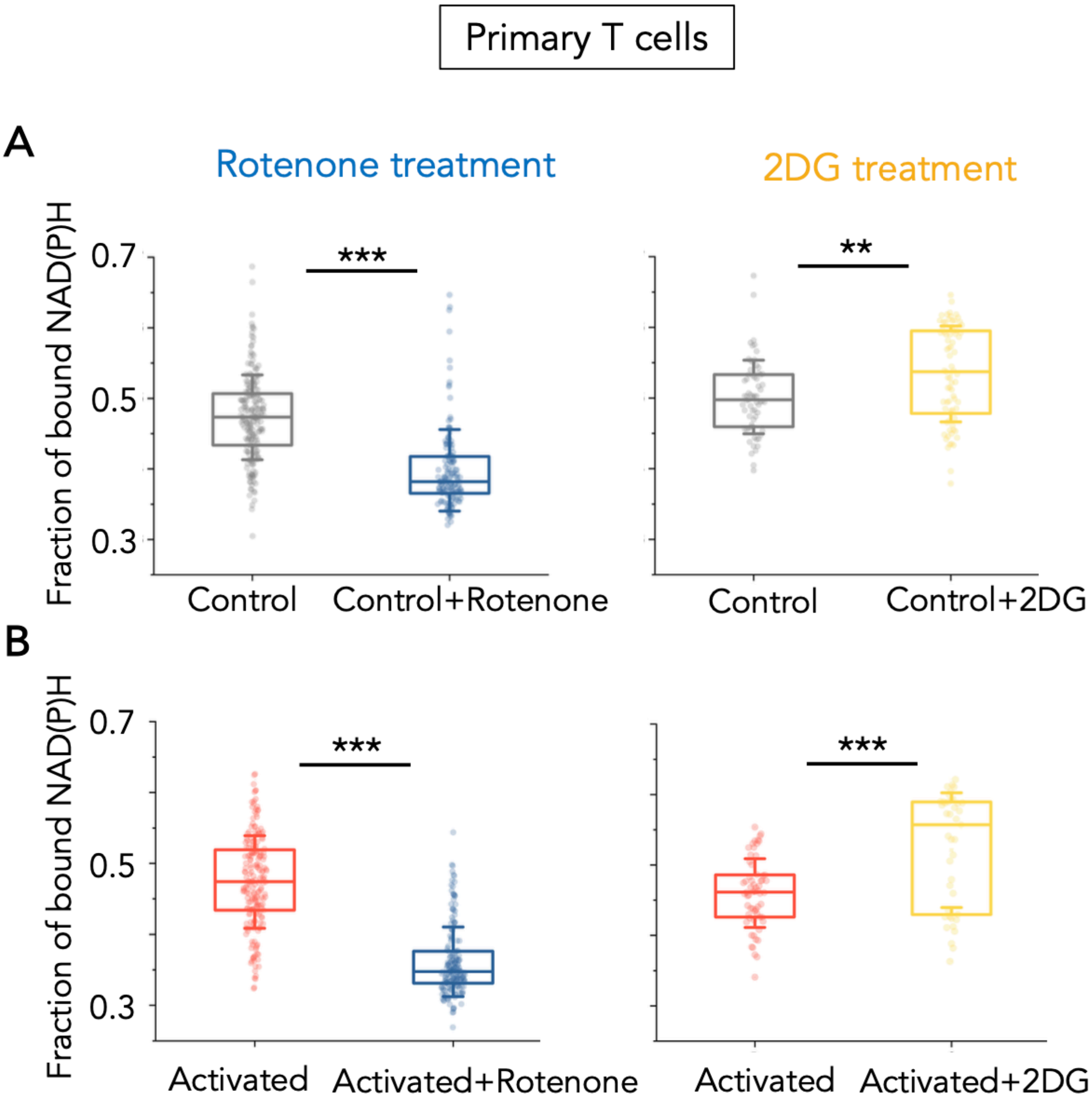

Figure S8: Metabolic trajectory in primary T cells following drug treatment. A. Quantification of fraction of bound NAD(P)H in primary T cells after rotenone (50 µM, 10 minutes) treatment in T cells from donor 2 or 2DG (10 mM, 30 minutes) treatment in T cells from donor 3 in control (non-activated) condition. **B.** Quantification of fraction of bound NAD(P)H in primary T cells after rotenone (50 µM, 10 minutes) treatment in T cells from donor 2 or 2DG (10 mM, 30 minutes) treatment in T cells from donor 3 in activated condition. Data are presented by mean with standard deviation range. T test: $^{**}P \leq 0.01$, $^{***} P \leq 0.001$

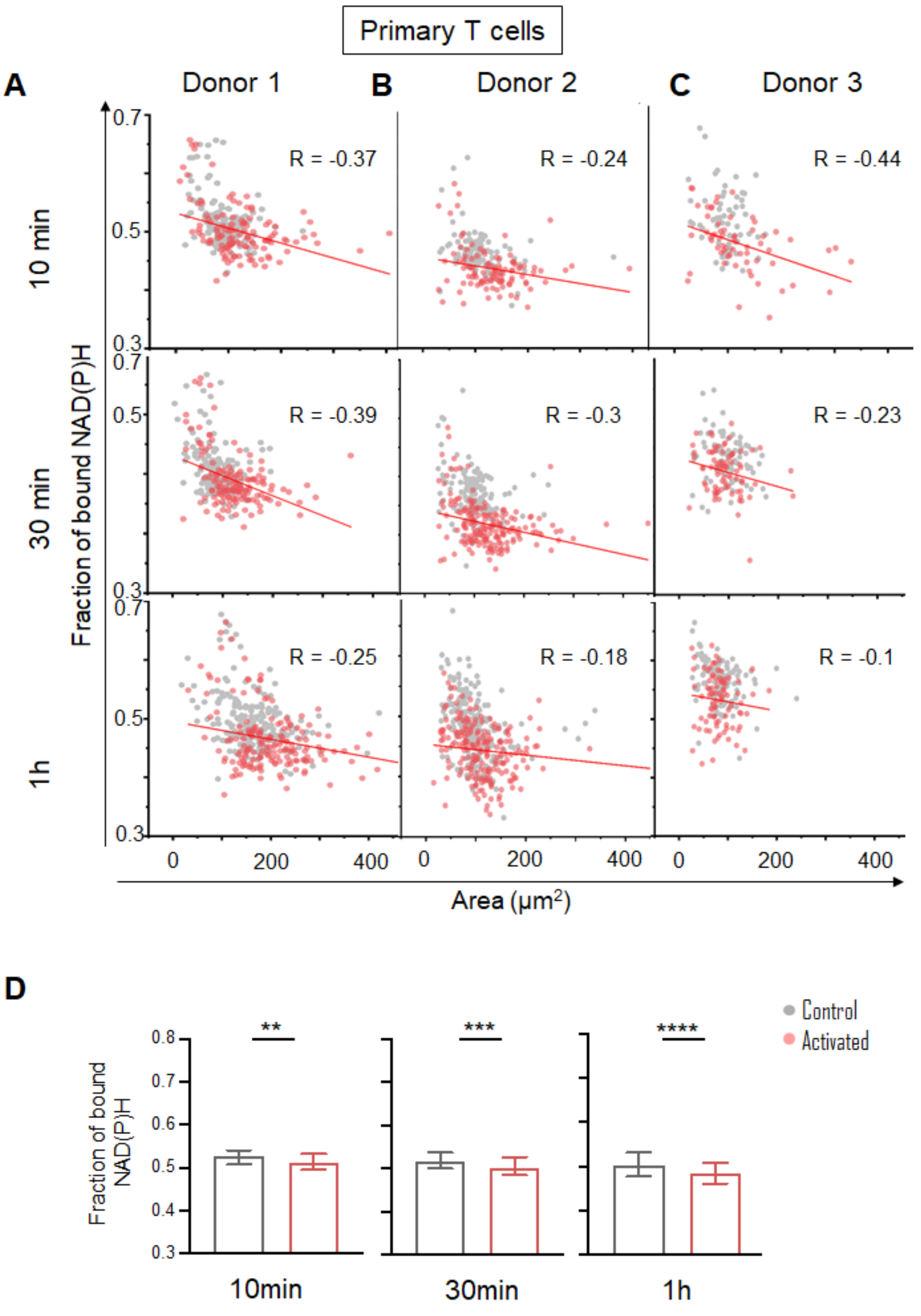

Figure S10

Figure S10: Simultaneous analysis of Primary T cell spreading and metabolic shift during activation, presented donor by donor. A-C. Correlation plots between cell areas and fraction of bound NAD(P)H of control and activated primary T cells over time (10 min, 30 min and 1 hour) for three donors. **D.** Representative quantification of the fraction of bound NAD(P)H over time (10 min, 30 min and 1 hour) in one donor (Donor 1 from figure 3). Data are presented by mean with standard deviation range. T test: **P ≤ 0.01, ***P<0.001, ****P<0.0001.

6. The authors should consider plotting the lifetime data too along with the bound fraction. This would make the results comparable to other studies/methods of lifetime analysis.

Answer: We added the figure of phase and modulation lifetime for both Jurkat T cells (fig. S5) and primary T cells (fig. S6). In the text we point out that, although it is possible to plot the NAD(P)H lifetime, in this paper we choose to plot the fraction of bound NAD(P)H because it is proportional to the redox ratio NAD(P)+/NAD(P)H.

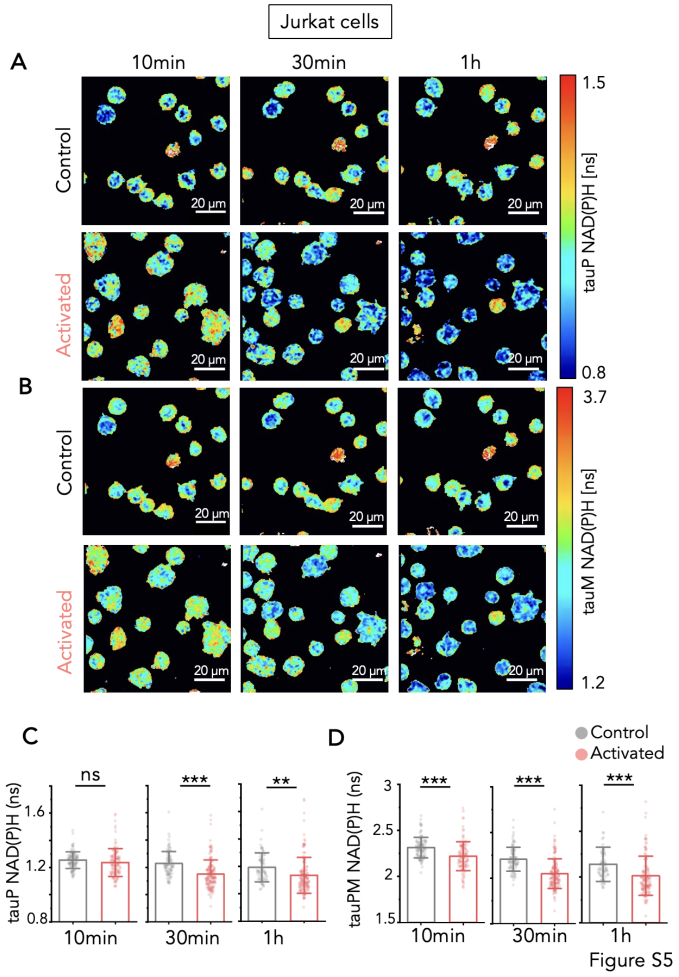

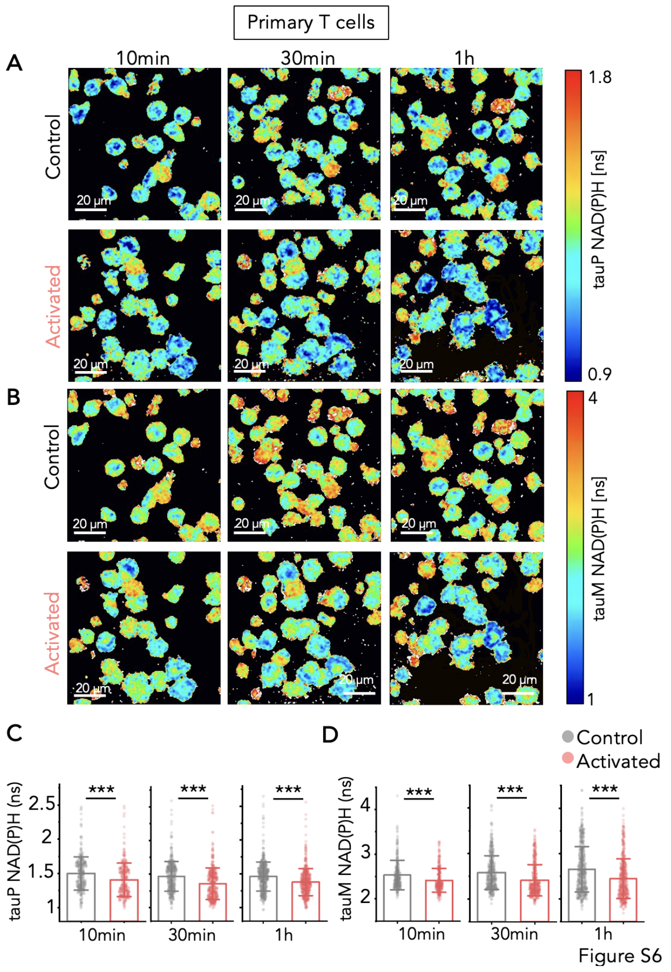

Figure S5: Metabolic shift during Jurkat T cell activation revealed by NAD(P)H FLIM. A. Representative images of phase lifetime (tauP) and **B.** modulation lifetime (tauM) of NAD(P)H of Jurkat T cells in control and activated condition at three time points (10 min, 30 min and 1 hour). Quantification of tauP (**C**) and tauM (**D**) of NAD(P)H over time of control and activated Jurkat T cells (10 min, 30 min and 1 hour). Each dot is one cell, data are presented by mean with standard deviation range. T-test: **P ≤ 0.01, ***P<0.001.

Figure S6: Metabolic shift during primary T cell activation revealed by NAD(P)H FLIM. A. Representative images of phase lifetime (tauP) and **B.** modulation lifetime (tauM) of NAD(P)H of primary T cells in control and activated condition at three time points (10 min, 30 min and 1 hour). Quantification of tauP (**C**) and tauM (**D**) of NAD(P)H over time of control and activated Jurkat T cells (10 min, 30 min and 1 hour). Data are presented by mean with standard deviation range. T test: ***P<0.001.N = 3 independent experiments with 3 different healthy donors. Data presented are means for single cells from the 3 donors. N = 3 independent experiments with 3 different healthy donors.

7. Explain what kind of z projection was done (Fig S1A)- summations/ maximum intensity..? Also, why was this chosen over creating separate mask for each z-plane

Answer: We performed a Z projection of the maximum intensity across all the Z planes of the 3D stack (Fig. S2A) on the LifeAct-mCherry or NAD(P)H intensity channel. We chose to define the masks to separate each individual cell using Z stacks over creating separate masks for each Z plane since the extreme planes (top and bottom) often had a very weak signal that did not allow the creation of a masks.

We added a paragraph in the materials and methods section (page 8) to better clarify the workflow used:

"We implemented single cell analysis to quantify the cellular mean of the fraction of bound NAD(P)H from the 3D FLIM data by using masks of single cells defined by the LifeAct-mCherry intensity for the stable cell line and by the NAD(P)H intensity for the primary T cells (Fig. S2A). First, we performed a maximum intensity Z-projection across all the Z planes of the 3D stack (Fig. S2A). Then, binary masks were created using a gaussian blur and a threshold to identify and segment each single cell (Fig. S2B) using Fiji-ImageJ (NIH, Bethesda, MD, USA). The same mask was used for every Z plane of one ROI rather than constructing separate masks for each Z plane. With a MATLAB custom written code, we also applied a threshold (5 photons) on the NAD(P)H intensity to consider only the cell signal and exclude the background at each Z plane. Then, using equation 3, we calculated the fraction of bound NAD(P)H maps (Fig. S1C) at every Z plane of the ROI by applying both the mask calculated on the maximum intensity Z-projection and the intensity threshold at every Z plane. Finally, to perform single cell segmentation, we applied the binary masks of single cells to the 3D stack fraction of bound NAD(P)H maps (Fig. S2C) and created 3D metabolic maps of single cells (Fig. S2D). We then calculated the average fraction of bound NAD(P)H for every Z plane of the cell and in the entire volume of the cell considering only the pixels above the intensity threshold."

8. Explain the steps for segmentation (eg was any thresholding performed?) for reproducibility.

Answer: We added a precise explanation of the steps used for segmentation in the Materials and methods section, part Analysis of the Fluorescence Lifetime microscopy:

"We implemented single cell analysis to quantify the cellular mean of the fraction of bound NAD(P)H from the 3D FLIM data by using masks of single cells defined by the LifeAct-mCherry intensity for the stable cell line and by the NAD(P)H intensity for the primary T cells (Fig. S2A). First, we performed a maximum intensity Z-projection across all the Z planes of the 3D stack (Fig. S2A). Then, binary masks were created using a gaussian blur and a threshold to identify and segment each single cell (Fig. S2B) using Fiji-ImageJ (NIH, Bethesda, MD, USA). The same mask was used for every Z plane of one ROI rather than constructing separate masks for each Z plane. With a MATLAB custom written code, we also applied a threshold (5 photons) on the NAD(P)H intensity to consider only the cell signal and exclude the background at each Z plane. Then, using equation 3, we calculated the fraction of bound NAD(P)H maps (Fig. S1C) at every Z plane of the ROI by applying both the mask calculated on the maximum intensity Z-projection and the intensity threshold at every Z plane. Finally, to perform single cell segmentation, we applied the binary masks of single cells to the 3D stack fraction of bound NAD(P)H maps (Fig. S2C) and created 3D metabolic maps of single cells (Fig. S2D). We then calculated the average fraction of bound NAD(P)H for every Z plane of the cell and in the entire volume of the cell considering only the pixels above the intensity threshold."

9. Consider plotting the variables of equation 3 on a phasor plot for easier understanding
Answer: We plotted the variables of equation 3 on the phasor plot of figure 1E and we also added the phasor plot color mapped with the fraction of bound NAD(P)H contrast (Figure 1F).

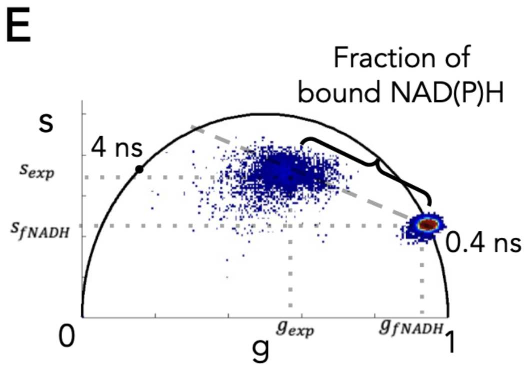

Figure 1: Experimental setup of *two-photon excitation Fluorescence Lifetime Imaging (FLIM).* E. The multi-exponential fluorescence intensity decay in every pixel of the image is transformed in the phasor plot by Fast Fourier transform. Phasor plot is color mapped as a histogram showing the density of points in phasor location. The estimation of the fraction of bound NAD(P)H is performed by measuring the distance of every experimental pixel from the location of free NAD(P)H. **F.** Phasor plot color mapped with the fraction of bound NAD(P)H contrast. Blue pixels in the right part of the phasor plot have low fraction of bound NAD(P)H values with a more glycolytic phenotype, while red pixels within the left part of the phasor plot have high fraction of bound NAD(P)H values and a more OXPHOS phenotype.

10. Fig 1B requires further explanation that what's provided in the legend else the authors could cite previous articles explaining the principle instead of including the diagram
Answer: We updated the figure 1B and provided a more detailed explanation in the legend.

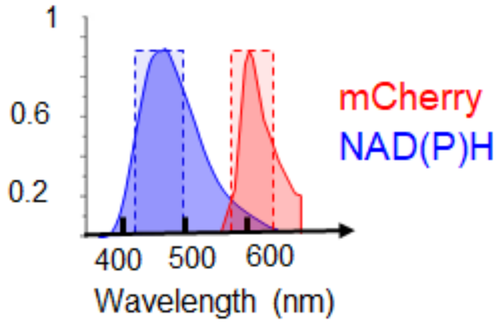

Figure 1: Experimental setup of *two-photon excitation Fluorescence Lifetime Imaging (FLIM).* B. Emission fluorescence of NAD(P)H and LifeAct-mCherry were collected with band-pass filters 460/80 and 607/70 respectively.

11. Authors mentioned that they did not observe any photodamage after longitudinal imaging. How was photodamage evaluated? Consider including the data.

Answer: We evaluated photodamage using a viability dye staining dead cells. This is now presented in figure S1. We observed very few dead cells during longitudinal FLIM imaging. In the data analysis of primary cells, we created a mask based on the viability dye channel and excluded these dead cells from the FLIM analysis. To reply to this point we added figure S1 and two paragraphs in the materials and methods section (page 6):

"We verified the negligible photo perturbation induced during our longitudinal imaging protocol by using a red viability dye (IncuCyteTM Cytotox Red Reagent, Cat no. 4632) during the FLIM imaging of NAD(P)H in primary cells (Fig S1). The red fluorescence of the viability dye is simultaneously collected with the NAD(P)H fluorescence through a red pass filter (Semrock FF01– 607/70). After 1 hour the T cells that were longitudinally imaged were still viable. In the statistical analysis of primary cells, we only considered viable cells while we excluded dead cells highlighted by the red viability dye (Fig. S1)."

and in the results section (page 9):

"We determined the minimal illumination conditions permitting longitudinal 2P-FLIM of NAD(P)H with a good signal to noise ratio and with negligible photo-perturbation (Material and Methods and Fig. S1)."

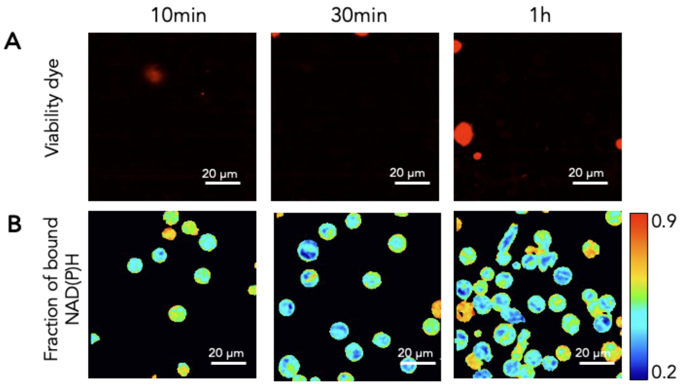

Figure S1: Assessing phototoxicity in primary T cells during longitudinal FLIM imaging of NAD(P)H using a viability dye. A. Representative images of dead cells stained with a viability dye in a ROI with primary T cells activated and imaged at 10min, 30min, and 1h during the 3D FLIM imaging protocol. After 1 hour the T cells that were longitudinally imaged were still viable. The dye was used to identify and exclude dead cells from the statistical analysis. **B.** Corresponding maps of fraction of bound NAD(P)H of the T cells from the same region of interest. We note that dead cells have a typical fraction of bound NAD(P)H that is higher than viable cells.

12. Authors should consider adding activation markers to their experiment to selectively identify cells that have been activated, for examples...

Answer: We have chosen to follow the metabolic trajectory in T cells activated for relatively short periods (10 minutes to 1 hour) because this timing has been less reported in the literature. This kinetic does not allow to follow classical activation markers such as CD69 or CD25, which take hours to be expressed by the T cells. This is also why we followed an early marker of activation, i.e., the spreading of the cells on activating substrates.

13. For the rotenone and 2DG experiments, author should include these controls: activated t cells+ rotenone and unstimulated +2DG

Answer: We included the activated T cells + rotenone and unstimulated cells + 2D controls for primary T cells and presented them in the new figure S8.

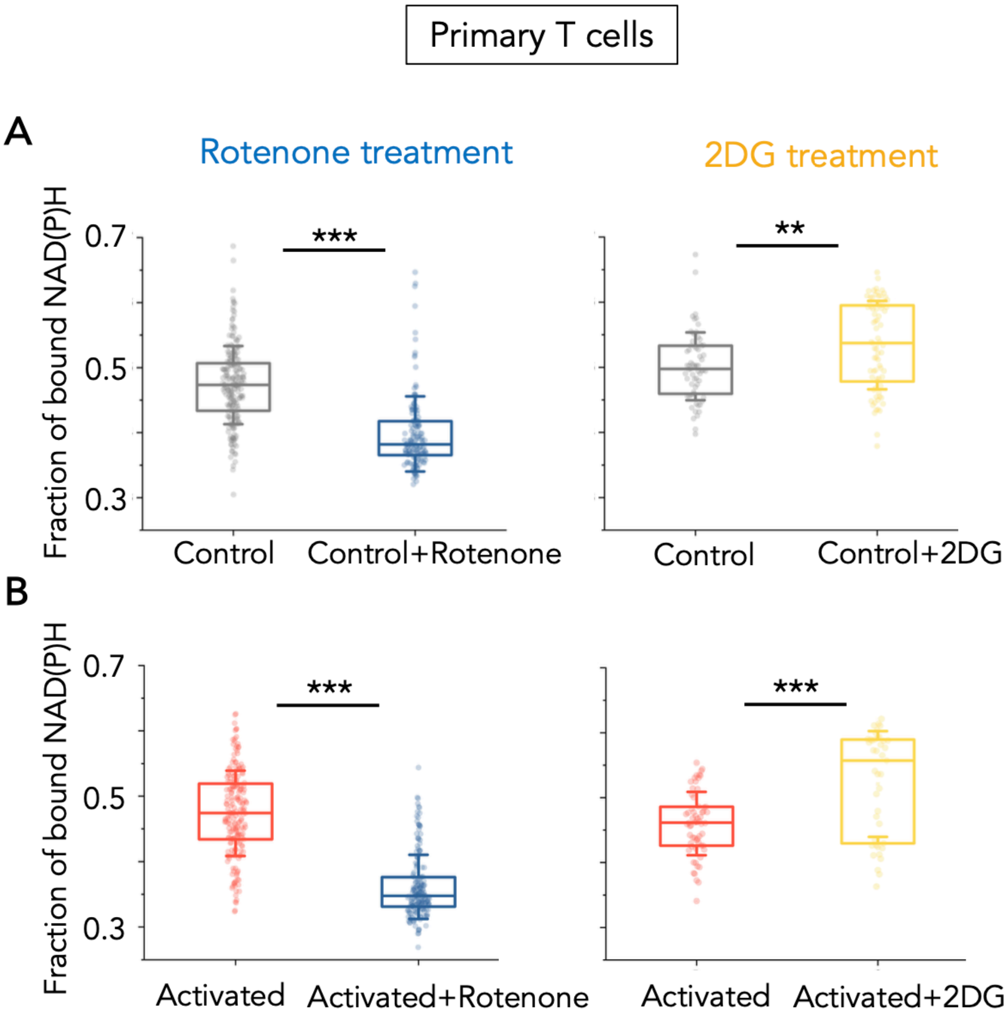

Figure S8: Metabolic trajectory in primary T cells following drug treatment. A. Quantification of fraction of bound NAD(P)H in primary T cells after rotenone (50 µM, 10 minutes) treatment in T cells from donor 2 or 2DG (10 mM, 30 minutes) treatment in T cells from donor 3 in control (non-activated) condition. **B.** Quantification of fraction of bound NAD(P)H in primary T cells after rotenone (50 µM, 10 minutes) treatment in T cells from donor 2 or 2DG (10 mM, 30 minutes) treatment in T cells from donor 3 in activated condition. Data are presented by mean with standard deviation range. T test: $**P \leq 0.01$, $*** P \leq 0.001$

14. For the 2DG experiment, were the cells preincubated with 2DG before activation? Please include details of these experiments in methods

Answer: Cells were preincubated 10 minutes with rotenone before imaging and 30 minutes with 2DG before activation and imaging. We included these details in the Materials and Methods section and the legends.

15. In the section, 'Monitoring fraction of bound NADH in single Jurkat...', authors mentions heterogeneity in previous paragraph but no heterogeneity analysis was performed.

Answer: As we did not quantify this heterogeneity, we removed this sentence from the section.

16. For 3D analysis, please plot the NADH intensity profile vs Z to confirm that the change is not driven by lack of photons. A zero cross correlation between the two would be a convincing affirmation.

Answer: Here below we plotted the mean intensity of NAD(P)H versus the mean fraction of bound NAD(P)H of the central Z planes (from 3um to 12um in figure 4A) of all single "control/non-activated" Jurkat T cells analyzed in Figure 2. The linear fitting is equal to R= -0.097, meaning that there is no significant correlation between NAD(P)H intensity and bound fraction of NAD(P)H. This confirms that indeed, the change in the fraction bound NAD(P)H in Z is not driven by a lack of photons.

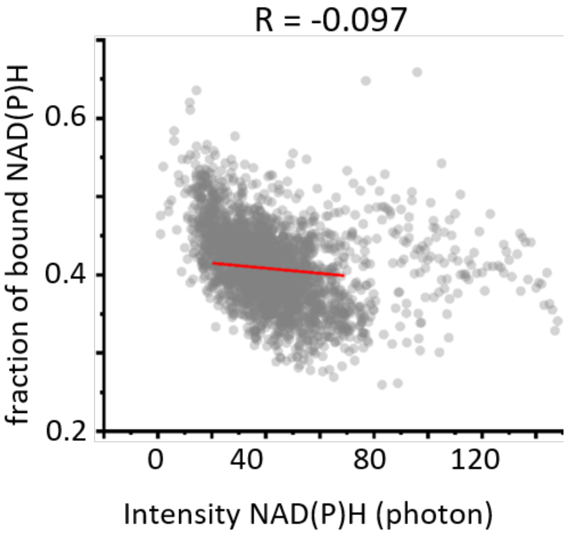

Three-dimensional intensity pattern in Jurkat T cell in control condition. We note that we excluded from the 3D analysis of Jurkat cells (Figure 4B and 4C) the Z planes at the edge of the cells (0um and 15um in figure 4A) as they contained only a few pixels with a sufficiently high number of photons.

17. To confirm that the authors are imaging mitochondria and nucleus at the depths that they are imaging, it would be convincing to image the cells with fluorescent markers for nucleus and mitochondria and perform a depth comparison

Answer: To confirm we are imaging mitochondria and nucleus, we stained the cells with DAPI (nucleus) and Mitotracker (mitochondria) and confirmed we can in fact identify these structures in the depths we are imaging. Representative images are shown in the new figure S9.

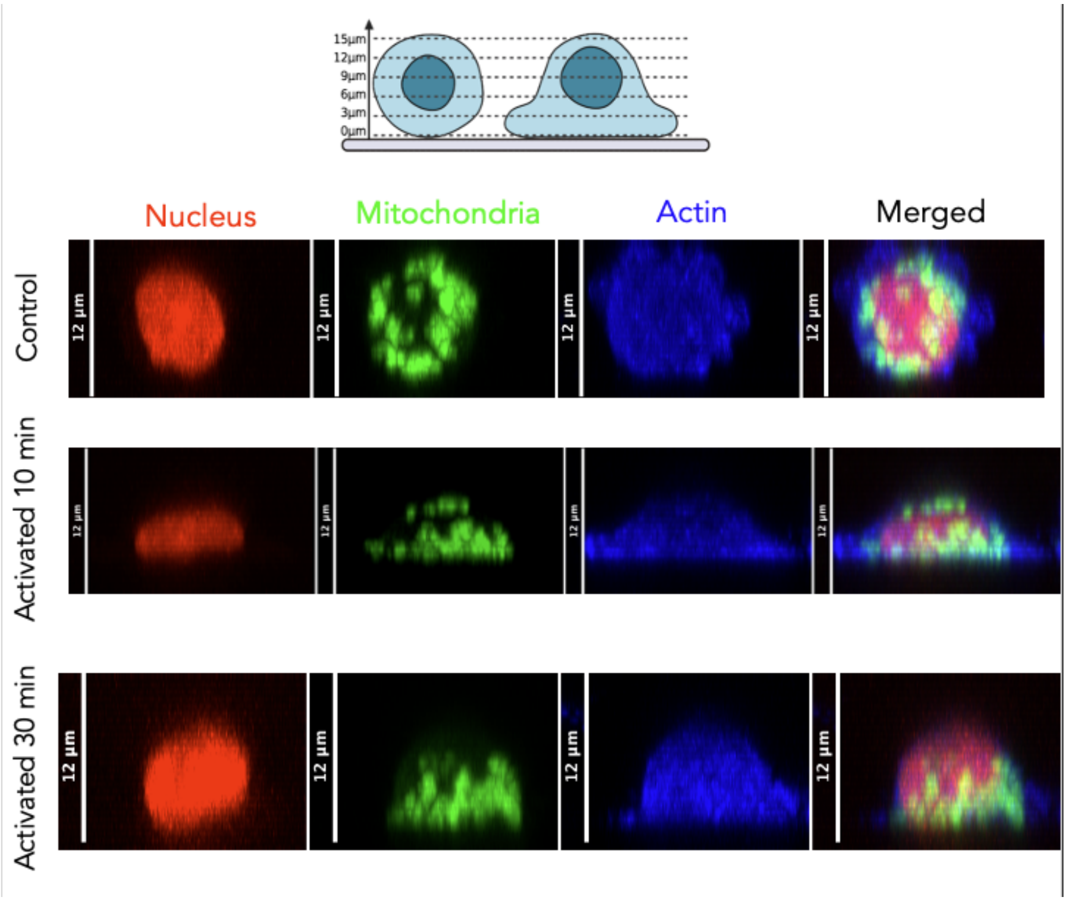

Figure S9: 3-dimensional (3D) visualization of mitochondria and nucleus in Jurkat T cells. Schematic presentation of the 3D imaging of Jurkat T cells and representative images of Jurkat T cells spreading on glass surfaces coated with poly-L-lysine (control) or poly-L-lysine and anti-CD3/CD28 antibodies (activated) for 10 min or 30 min. The actin was visualized with LifeAct-mCherry, mitochondria were stained prior fixation with Mitotracker and the nucleus was stained after fixation and permeabilization with DAPI. Images were acquired with a confocal microscope and Z stacks were reconstituted to visualize mitochondria and nucleus distribution across the cell in each condition.

18. For the LifeAct-mCherry experiment, could the authors perform a time lapse analysis mCherry signal from 0min to 10 or more to show significant increase at 10 mins?
Answer: We could not perform a time lapse analysis of the mCherry signal from 0 to 10mins, since this induced the bleaching of the mCherry and thus a decrease of the signal.

Minor:

1. In the abstract mention the subtype of T cells used in this study
Answer: We have now added the type of cells used in the study in the abstract, as well as in the main text and figures.

2. Introduction, second paragraph, 2nd line'...we can cite..., and 4th line ' we can also cite..' please consider rephrasing this.
Answer: We removed this repetition from the introduction.

3. Section: analysis of images, 1st line in 1st paragraph, '..treated with Fiji-ImageJ.." what does that mean?
Answer: We clarified this sentence and replaced it by "Intensity data were obtained using Fiji-ImageJ (NIH, Bethesda, MD, USA) by doing maximum intensity projections of all stacks of FLIM images (Fig. S2)."

4. The title of the 2nd results ..'upon time' does not make sense
Answer: We changed the title from "upon time" to "over time"

5. Ref 12 and 24 are the same
Answer: We removed the reference number 24.

Reviewer #2:

Paper review

In general, the authors applied the two-photon fluorescence lifetime microscopy to detect the spatial and temporal metabolic perturbations of T-cells upon activation by measuring NADH fluorescence lifetime. They found that the T-cell activation led to a decrease in bound NADH fraction after 10 minutes, which implied a shift towards glycolysis. Additionally, three-dimensional mapping of the NADH bound fraction revealed different NADH bound fractions in different cellular compartments, and asymmetry distribution in activated T-cells between the cell side in contact with the slide and its opposite, which did not appear in resting T-cells.

1. Introduction

In the introduction, the authors first introduced the importance of T-cell metabolism, current modalities of measuring metabolism in T lymphocytes, and their limitations. Then, the authors put forward the twophoton fluorescence lifetime microscopy to detect NADH to address metabolic variations. Finally, the authors generally introduce the work and results of this paper. NADH is an electron acceptor in glycolysis and an electron donor in oxidative phosphorylation. Therefore, on page 4, I suggested rewriting this sentence "NADH is the principal electron acceptor in glycolysis in oxidative phosphorylation and its ubiquity renders NADH one of the most useful and informative intrinsic biomarkers for metabolism in live cells and tissues.
Answer: Since indeed this sentence was unclear, we changed it for "NAD(P)H fluorescence lifetime is exquisitely sensitive to enzyme binding during the cycling of the electron transport chain, the protein-bound NAD(P)H lifetime being significantly longer than the free NAD(P)H lifetime. Both this particularity and its ubiquity render NAD(P)H one of the most useful and informative intrinsic biomarkers for metabolism in live cells and tissues [16,17]."

2. Materials and methods

In the materials and methods, the authors introduced the details of cell preparation, cell activation, imaging procedure, and fluorescence lifetime analysis. Here are the questions for the methods section: I am confused about the acquisition time of a 3D FLIM image. The 3D FLIM image was composed of 6 2D FLIM images. However, it took around one time to get a 2D FLIM, but why did it just take around 2 minutes to take a 3D FLIM image? The time is much shorter than the theoretical time, did you change the imaging parameters, can you please clarify this?
Answer: We explained in detail the acquisition time of the 3D FLIM in the Materials and methods section, part Two-photon excited fluorescence lifetime imaging (FLIM). It takes 21.86s to acquire one FLIM image. So one 3D FLIM data including 6 single FLIM images takes 131s with a typical pixel dwell time of 334 us/pixel. We actually reported an incorrect value in the first submission of the paper.

We added the following paragraph at page 6:

"We typically collected 200-300 photons during an acquisition time in the order of seconds for a 256x256 pixels image at a pixel dwell time of 334 µs/pixel. The three-dimensional (3D) FLIM

imaging was performed with a Z-step of 3 µm and the total acquisition time of a 3D FLIM with 6 Z planes is in the order of two minutes."

For the sentence" Intensity data were analyzed and treated with Fiji-ImageJ (NIH, Bethesda, MD, USA)." It is not clear what analysis you performed, and treated with ImageJ. Can you please provide more details about that?
Answer: We provided more details about the image analysis workflow, and we inserted the following paragraph:

"We implemented single cell analysis to quantify the cellular mean of the fraction of bound NAD(P)H from the 3D FLIM data by using masks of single cells defined by the LifeAct-mCherry intensity for the stable cell line and by the NAD(P)H intensity for the primary T cells (Fig. S2A). First, we performed a maximum intensity Z-projection across all the Z planes of the 3D stack (Fig. S2A). Then, binary masks were created using a gaussian blur and a threshold to identify and segment each single cell (Fig. S2B) using Fiji-ImageJ (NIH, Bethesda, MD, USA). The same mask was used for every Z plane of one ROI rather than constructing separate masks for each Z plane. With a MATLAB custom written code, we also applied a threshold (5 photons) on the NAD(P)H intensity to consider only the cell signal and exclude the background at each Z plane. Then, using equation 3, we calculated the fraction of bound NAD(P)H maps (Fig. S1C) at every Z plane of the ROI by applying both the mask calculated on the maximum intensity Z-projection and the intensity threshold at every Z plane. Finally, to perform single cell segmentation, we applied the binary masks of single cells to the 3D stack fraction of bound NAD(P)H maps (Fig. S2C) and created 3D metabolic maps of single cells (Fig. S2D). We then calculated the average fraction of bound NAD(P)H for every Z plane of the cell and in the entire volume of the cell considering only the pixels above the intensity threshold."

3. Results

In this paper, the authors have three main findings. Firstly, there was a reduction of bound NADH fraction in T-cells after activation. Secondly, three-dimension imaging of the NADH-bound fraction revealed temporal and spatial changes in T-cells after activation. Finally, morphology changes were observed by fluorescence imaging of LifeAct-mCherry and NADH. Here are the suggestions and questions for the result parts:
In Figures 1D and 1F, can you provide the scale bar size for each image?
Answer: We added the scale bars and sizes in all the figures.

In Figure 1E, what does the colorbar mean? Did it mean the NADH bound fraction or the density of the clusters in the phasor plot?
Answer: To clarify this point, we plotted separately the phasor plot as a histogram of the density of the pixels (Fig1E) and the phasor plot color mapped with the fraction of bound NAD(P)H contrast (Fig 1F). We also added relative explanations both in the text and in the figure captions.

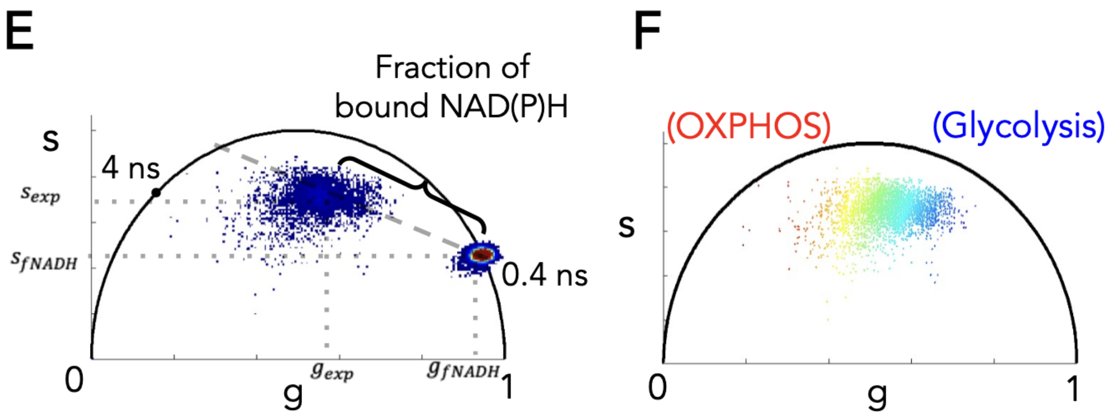

Figure 1: Experimental setup of *two-photon excitation Fluorescence Lifetime Imaging (FLIM).* E. The multi-exponential fluorescence intensity decay in every pixel of the image is transformed in the phasor plot by Fast Fourier transform. Phasor plot is color mapped as a histogram showing the density of points in phasor location. The estimation of the fraction of bound NAD(P)H is performed by measuring the distance of every experimental pixel from the location of free NAD(P)H. **F.** Phasor plot color mapped with the fraction of bound NAD(P)H contrast. Blue pixels in the right part of the phasor plot have low fraction of bound NAD(P)H values with a more glycolytic phenotype, while red pixels within the left part of the phasor plot have high fraction of bound NAD(P)H values and a more OXPHOS phenotype.

In the sentence" In the presence of anti-CD3+CD28 antibodies, the 2P fluorescence intensity of NADH revealed a more even distribution close to the activating slide, reflecting changes in the morphology of activated T cells with a possible accumulation of mitochondria at the contact zone (surrogate immune synapse)." Can you please provide the attached result image?

Answer: We have now removed this sentence since this was not corroborated by quantification of the distribution of the mitochondria in the images. Yet, in the 3-dimensional analysis of the fraction of bound NAD(P)H (new figure 4), we propose that changes between top and bottom of the cell is due to the distribution of mitochondria in the cell and the localization of the nucleus. We are now more cautious in the text and show representative images of the localization of mitochondria and nucleus in resting and activated cells (new figure S9).

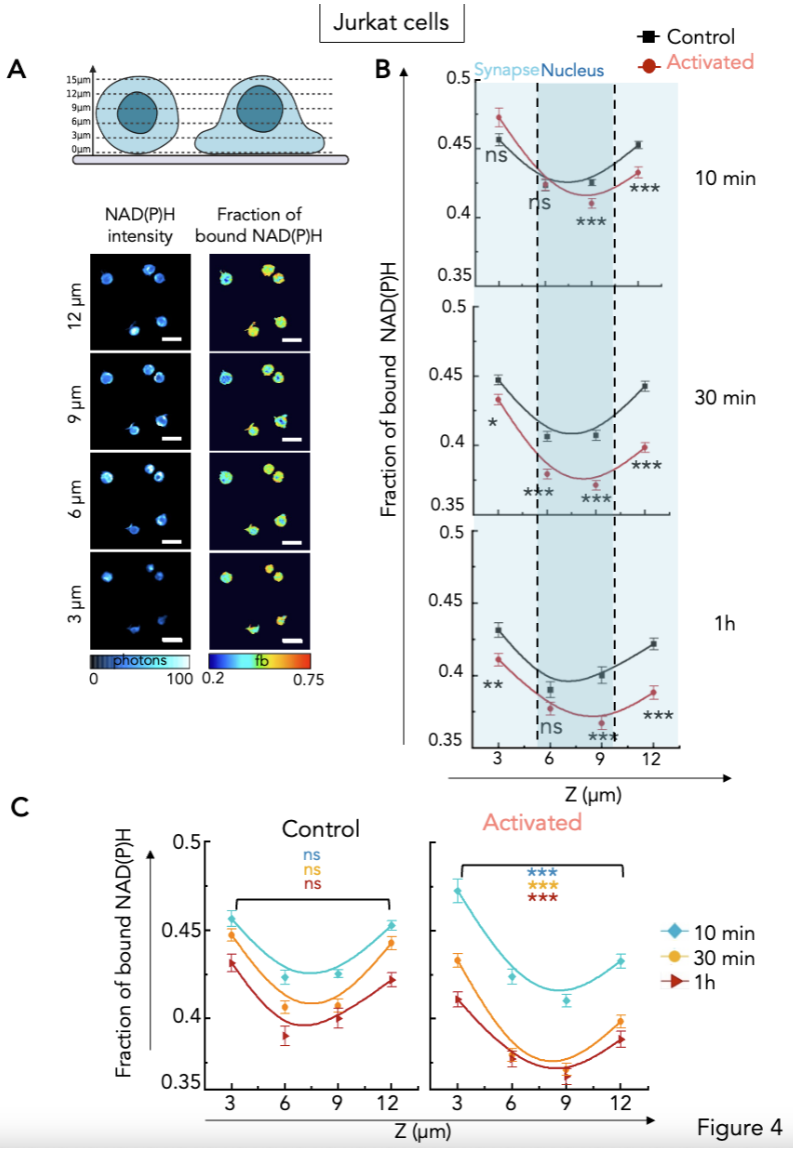

Figure 4: Three-dimensional analysis of the fraction of bound NAD(P)H in control and activated Jurkat cells. A. Schematic representation of 3D imaging performed in T cells and representative images of NAD(P)H intensity and fraction of bound NAD(P)H of single Jurkat T cells in the control condition at 10 mins in different Z planes. B-C. Quantification of fraction of bound NAD(P)H in control and activated conditions in every Z plane of the cells at 3 time points: 10 min, 30 min and 1 hour. B. Comparison of the intracellular distribution of the fraction of bound NAD(P)H between non activated (control) and activated Jurkat T cells at each time point. Data are presented as mean with standard deviation range. Statistics are indicated for each timepoint, and the 4 Z planes analyzed. **C. C**omparison of the fraction of bound NAD(P)H between bottom and top of the cell in non-activated (control) and activated Jurkat T cells. Statistics between Z=3µm and Z= 12µm are indicated for each time point in a different color. Data are presented as mean with standard deviation range. T test: $**P \leq 0.01$, $*** \leq 0.001$, ns: non-significant.

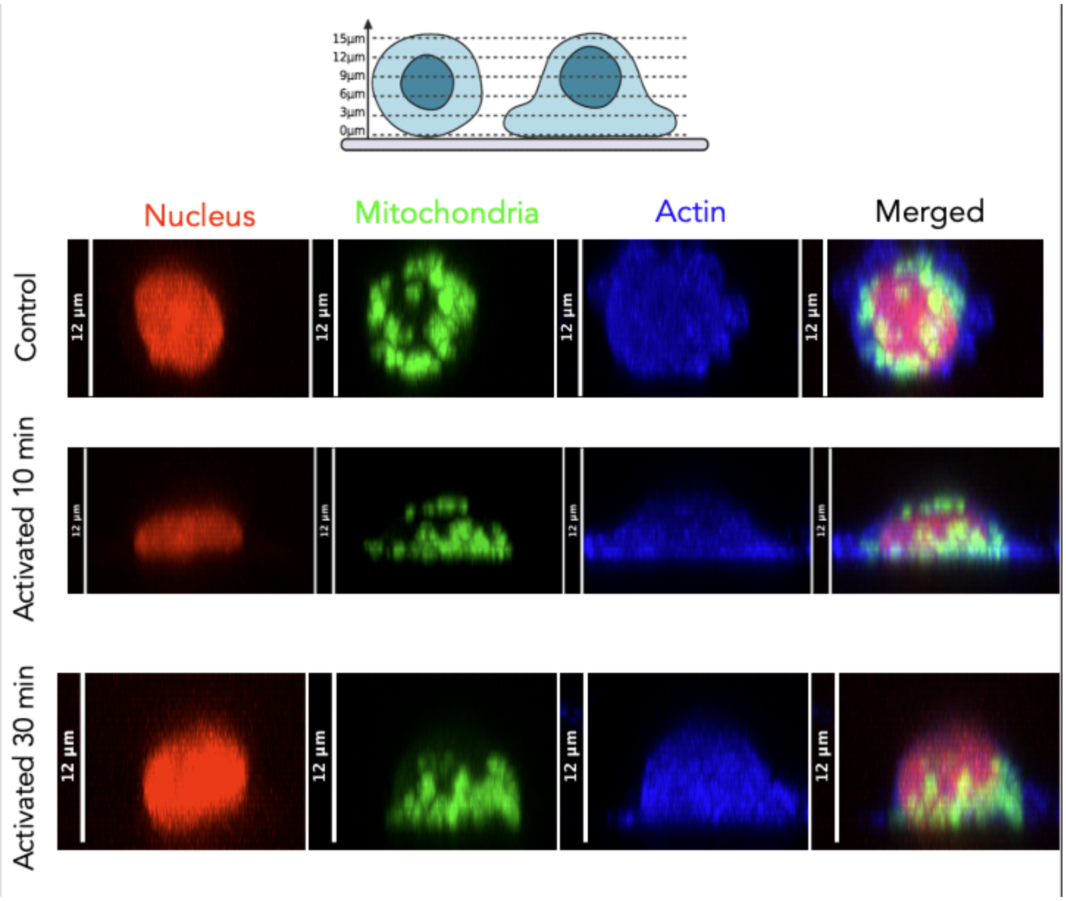

Figure S9: 3-dimensional (3D) visualization of mitochondria and nucleus in Jurkat T cells. Schematic presentation of the 3D imaging of Jurkat T cells and representative images of Jurkat T cells spreading on glass surfaces coated with poly-L-lysine (control) or poly-L-lysine and anti-CD3/CD28 antibodies (activated) for 10 min or 30 min. The actin was visualized with LifeAct-mCherry, mitochondria were stained prior fixation with Mitotracker and the nucleus was stained after fixation and permeabilization with DAPI. Images were acquired with a confocal microscope and Z stacks were reconstituted to visualize mitochondria and nucleus distribution across the cell in each condition.

Also, I am not convinced by the result that more even distribution is close to the activating slide in Figure 2. Can you please explain this or provide a statistical comparison result?
Answer: As stated above we removed the sentence claiming more even distribution of 2P fluorescence NAD(P)H intensity close to the activating slide.

I can find a lower fB NADH in the control (1h) compared to the control (30min), so to make the result more comprehensive, I suggest combing the three figures in Figure 3D into one figure and adding a statistical comparison in the control and activated groups respectively to compare the variations of NADH bound fraction over time.

Answer: We now added a supplemental figure (new figure S4) showing the variation of the fraction of bound NAD(P)H in non-activated (control) and activated cells overtime. Although, as stated by the reviewer the values are decreasing in time, they are significantly different between control and activated conditions for each time point (figure 2D). Thus, we can conclude that activation of T lymphocytes induces a decrease of fraction of bound NAD(P)H in the time scale that we have covered.

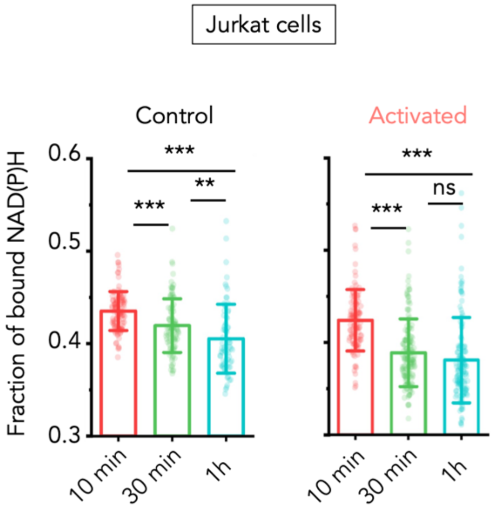

Figure S4: Longitudinal analysis of the fraction of bound NAD(P)H in non-activated (Control) and activated Jurkat T cells. A. Quantification of fraction of bound NAD(P)H of Jurkat T cells over time (10 min, 30 min and 1 hour) in control and activated condition. Each dot represents one cell. Mean with standard deviation range are shown. T test: $^{**}P \leq 0.01$, $^{***}P<0.001$. N= 2 experiments.

The R-value between area and fB NADH is below 0.1, so it was hard to conclude that there was a negative relationship. I suggest changing Figure 5C and rewriting the relevant sentences in the results and discussion.

Answer: Indeed, the negative correlation between the spreading area of Jurkat T cells and the fraction of bound NAD(P)H has a R-value<-0,1 at time 30 minutes. Yet, the R-values at 10 minutes and 1H are >-0.1 for Jurkat cells. The negative correlation between the spreading area and the fraction of bound NAD(P)H drawn from the data obtained in primary T cells are more convincing with R-values around -0.3. These new data are shown in the new figure 6 and new supplemental figure S10. We have now been more cautious in the text in the interpretation using the term s tendency rather than negative correlation.

4. Discussion

In the discussion part, the authors mainly summarized the work of this paper, compared the results with the previous studies, and hypothesized the spatial differences of NADH bound fraction was caused by the immune synapse. It seems that the NADH intensity images can provide information about cell spreading size, and the authors could reproduce the results in primary T cells (Figure S3). What is the advantage of using mCherry over NADH intensity and what other information can mCherry tell? I suggest the authors provide more discussions about this.
Answer: The advantage of imaging the LifeAct-mCherry is to be able to follow F-actin remodeling together with metabolic changes. We clarify this point in the discussion.

Some minor comments: Can you please reference for the sentence “while most of the studies analyze metabolic changes after 24h to 48h of activation” in the discussion.
Answer: We have now added references for this sentence.

Can you provide the scale bar size for all the images?
Answer: We now added all the scale bars and sizes in the figures.

In Figure S1, can you please change the intensity image to make them match the other images?
Answer: This has been done.

Some typos: FFT needs a full form; Matlab should be MATLAB
Answer: Typos have been corrected.

References:

1. E. Sánchez-Ramírez, T. P. L. Ung, A. Alarcón Del Carmen, X. Del Toro-Ríos, G. R. Fajardo-Orduña, L. G. Noriega, V. A. Cortés-Morales, A. R. Tovar, J. J. Montesinos, R. Orozco-Solís, C. Stringari, L. Aguilar-Arnal, Coordinated metabolic transitions and gene expression by NAD+ during adipogenesis. *Journal of Cell Biology*. **221** (2022), doi:10.1083/JCB.202111137/213521.

RE: Manuscript #E23-01-0009R
TITLE: "Label-free single-cell live imaging reveals fast metabolic switch in T lymphocytes"

Dear Dr. Hivroz:

I am pleased to accept your manuscript for publication in Molecular Biology of the Cell.

The authors have satisfactorily addressed the reviewers. The authors should ensure that the manuscript meets the requirements laid out in the MBoC Author Submission Checklist, including indicating the number of samples (or cells as appropriate) analyzed in each figure etc.

Sincerely,
Avery August
Monitoring Editor
Molecular Biology of the Cell

Dear Dr. Hivroz:

Congratulations on the acceptance of your manuscript! Thank you for choosing to publish your work in Molecular Biology of the Cell (MBoC).

Within 10 days, an unedited PDF of your manuscript will be published on MBoC in Press, an early release version of the journal. The date your manuscript appears on this site is the official publication date.

Your copyedited and typeset manuscript will be scheduled for publication in the next available issue of MBoC. Our production team will notify you when the page proofs of your paper are ready for your review.

MBoC offers the option to publish your paper with immediate open access. Open access can increase the discoverability and usability of your research. If you would like to publish your paper with immediate open access but did not select this option during initial submission, please contact the MBoC Editorial Office (mbc@ascb.org).

We invite you to submit images related to your accepted manuscript to be considered for the journal cover. Please contact mboc@ascb.org to learn how to submit candidate cover images.

Authors of Articles and Brief Communications are also encouraged to create a short video abstract to accompany their article when it is published. These video abstracts, known as Science Sketches, are up to 2 minutes long and will be published on YouTube and then embedded in the article abstract. Science Sketch Editors on the MBoC Editorial Board will provide guidance as you prepare your video. Information about how to prepare and submit a video abstract is available at www.molbiolcell.org/science-sketches. Please contact mboc@ascb.org if you are interested in creating a Science Sketch.

We look forward to publishing your paper in MBoC.
Sign up for MBoC's new content alerts here https://tinyurl.com/3bcsyk3e

Sincerely,

Eric Baker
Journal Production Manager
MBoC Editorial Office
mbc@ascb.org

Reviewer #1 (Remarks to the Author):

The response to the review is satisfactory and the manuscript has improved considerably.

Reviewer #2 (Remarks to the Author):

The authors applied the two-photon fluorescence lifetime microscopy to detect the spatial and temporal metabolic perturbations of T-cells upon activation by measuring NADH fluorescence lifetime.
